# Supplementary material for: IDH1 R132 mutations or HER2-positivity and benefit from platinum-based therapy for biliary tract cancers
Source: JHEP Rep. 2026 May 20;8(8):101899. doi: 10.1016/j.jhepr.2026.101899 (PMC13380706; doi:10.1016/j.jhepr.2026.101899)
Supplement: Multimedia component 1 [file mmc1.pdf]

IDH1 R132 mutations or HER2-positivity and benefit from platinum-based therapy for biliary tract cancers

Giulia Tesini, Jack Greaves, Olivia Knight, Holly Shand, Angela Ammirabile, Sophie McHaffie, Jen Milne, Halima Ibrahim, Rosemary Meenan, Sophia Campbell, Margaret Henderson, Andrea Lampis, Louis Chesler, Andrea Casadei-Gardini, Lorenza Rimassa, Alan Christie, Timothy J Kendall, Chiara Braconi

Table of contents

Fig. S1……………………………………………………………………………………….2

Fig. S2……………………………………………………………………………………….3

Fig. S3……………………………………………………………………………………….4

Table S1………………………………………………………………..……..separate excel file

Table S2…………………………………………………………………….…...separate excel file

Table S3………………………………………………………………..……..separate excel file

Table S4……………………………………………………………………………………5

Supplementary figures

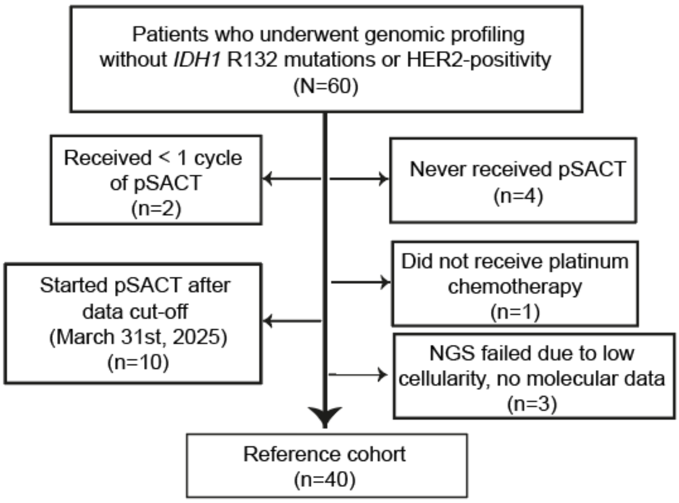

Fig. S1. CONSORT diagram of patients included in the reference cohort.

Abbreviations: NGS, Next-Generation Sequencing; pSACT, palliative systemic anti-cancer treatment.

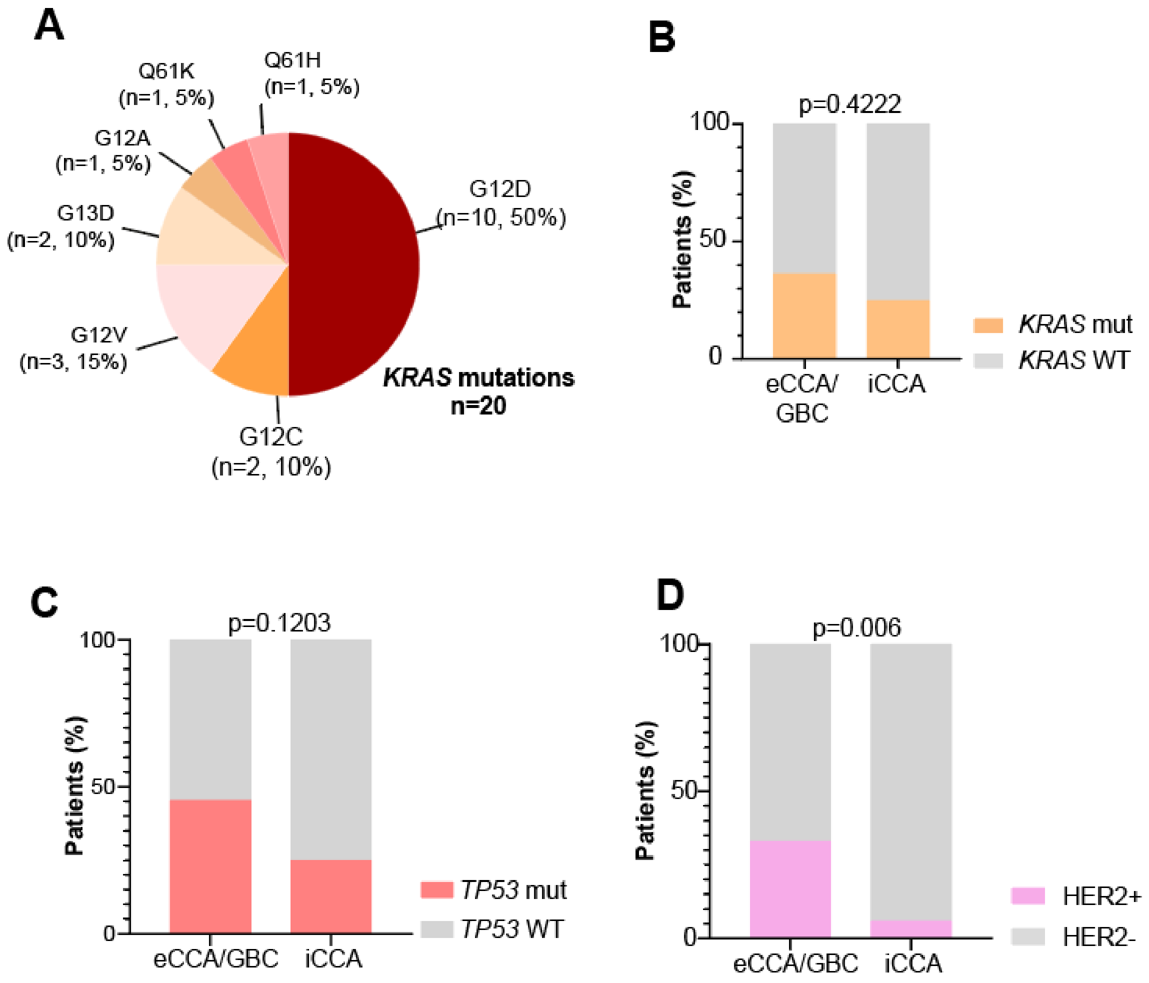

Fig. S2. Frequency of *KRAS* mutations (A) and distribution of the most common molecular alterations in our cohort across BTC subtypes (*KRAS* mutations – B; *TP53* alterations – C; HER2 overexpression (IHC 3+) or amplification (FISH-positive) – D). Variables have been compared using Fisher's exact test.

Abbreviations: eCCA, extrahepatic cholangiocarcinoma; GBC, gallbladder cancer; iCCA, intrahepatic cholangiocarcinoma; mut, mutated; WT, wild-type.

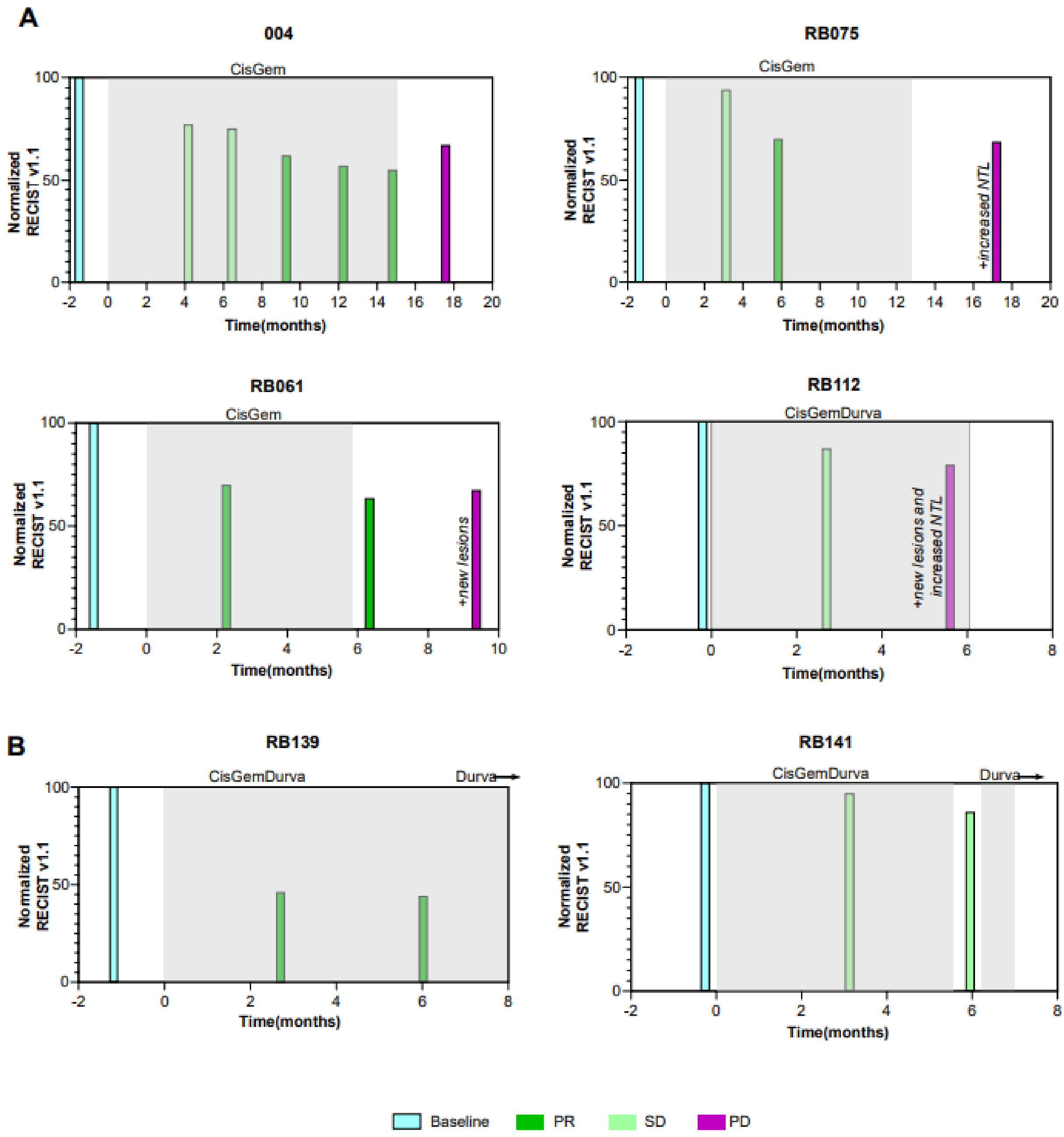

Fig. S3. Radiological response to treatment according to RECIST v1.1 criteria in patients with IDH1 R132 mutations. (A) Patients who have already progressed to first-line pSACT. (B) Patients who are still on treatment as of September 30th, 2025 (data cut-off). Radiological response improves over time and maximum radiological response is achieved > 5 months from treatment start in 5 cases. Abbreviations: CisGemDurva, cisplatin-gemcitabine-durvalumab; NTL, non target lesion; PD, progressive disease; PR, partial response; pSACT, palliative systemic anti-cancer treatment; SD, stable disease.

Supplementary tables

Table S1, S2 and S3 are provided in a separate Excel file.

Table S4. Baseline characteristics of the reference cohort.

	**N=40**
**Female, n(%)**	16 (40)
**Median age at diagnosis, years (IQR)**	62 (57-72)
**BTC subtype, n(%)**	
iCCA	19 (47.5)
pCCA	8 (20)
dCCA	6 (15)
GBC	7 (17.5)
**Resectable disease at diagnosis, n(%)**	12 (30)
**First-line pSACT, n(%)**	
Platinum/gemcitabine	14 (35)
Platinum/gemcitabine/durvalumab	26 (65)
**Median CA19-9 at start of 1L pSACT, kU/L (IQR)**	280 (33.5-3665)
**Median CEA at start of 1L pSACT, ug/L (IQR)***	3.5 (2.05-11.5)

*CEA at baseline not dosed for 3 patients

Abbreviations: 1L, first line; BTC, biliary tract cancer; CA19-9, carbohydrate antigen 19-9; CEA, carcinoembryonic antigen; dCCA, distal cholangiocarcinoma; GBC, gallbladder cancer; iCCA, intrahepatic cholangiocarcinoma; IQR, interquartile range; pCCA, perihilar cholangiocarcinoma; pSACT, palliative systemic anti-cancer treatment.
