# Supplementary material for: IDH1 R132 mutations or HER2-positivity and benefit from platinum-based therapy for biliary tract cancers
Source: JHEP Rep. 2026 May 20;8(8):101899. doi: 10.1016/j.jhepr.2026.101899 (PMC13380706; doi:10.1016/j.jhepr.2026.101899)
Supplement: Multimedia component 2 [file mmc2.docx]

**JHEP Reports**

**CTAT methods**

Tables for a “Complete, Transparent, Accurate and Timely account” (CTAT) are now mandatory for all revised submissions. The aim is to enhance the reproducibility of methods.

- Only include the parts relevant to your study
- Refer to the CTAT in the main text as ‘Supplementary CTAT Table’
- Do not add subheadings
- Add as many rows as needed to include all information
- Only include one item per row

**If the CTAT form is not relevant to your study, please outline the reasons why:**

|  |
| --- |

- 1. **Antibodies**

| **Name** | **Citation** | **Supplier** | **Cat no.** | **Clone no.** |
| --- | --- | --- | --- | --- |
|  |  |  |  |  |

- 1. **Cell lines**

| **Name** | **Citation** | **Supplier** | **Cat no.** | **Passage no.** | **Authentication test method** |
| --- | --- | --- | --- | --- | --- |
|  |  |  |  |  |  |

- 1. **Organisms**

| **Name** | **Citation** | **Supplier** | **Strain** | **Sex** | **Age** | **Overall n number** |
| --- | --- | --- | --- | --- | --- | --- |
|  |  |  |  |  |  |  |

- 1. **Sequence based reagents** (see below for gene fragments)

| **Name** | **Sequence** | **Supplier** |
| --- | --- | --- |
| ddPCR Mutation Assay:IDH1 p.R132S  (c.394C>A), Human  Unique Assay  ID:dHsaMDV2516816 | Wild Type (*c.394C)* Probe:  *Proprietary Information*  *Mutant (c.394A) Probe:*  *Proprietary Information* | Bio-Rad Laboratories |
| ddPCR Mutation Assay:IDH1 p.R132L,  Human  Unique Assay ID: dHsaMDV2516818 | Wild Type (*c.395G)* Probe:  *Proprietary Information*  *Mutant (c.395T) Probe:*  *Proprietary Information* | Bio-Rad Laboratories |
| ddPCR Mutation Assay:IDH1 p.R132G,  Human  Unique Assay ID: dHsaMDV2510512 | Wild Type (*c.394C)* Probe:  *Proprietary Information*  *Mutant (c.394G) Probe:*  *Proprietary Information* | Bio-Rad Laboratories |
| ddPCR Mutation Assay:IDH1 p.R132C,  Human  Unique Assay ID: dHsaMDV2010053 | Wild Type (*c.394C)* Probe:  *Proprietary Information*  *Mutant (c.394T) Probe:*  *Proprietary Information* | Bio-Rad Laboratories |

- 1. **Biological samples**

| **Description** | **Source** | **Identifier** |
| --- | --- | --- |
|  |  |  |

- 1. **Deposited data**

| **Name of repository** | **Identifier** | **Link** |
| --- | --- | --- |
|  |  |  |

- 1. **Software**

| **Software name** | **Manufacturer** | **Version** |
| --- | --- | --- |
| ddPCR: QuantaSoft | Bio-Rad | 2.2 |
| Statistical analysis: GraphPad Prisms | Dotmatics | 10.5.0 |

- 1. **Other (*e.g*. drugs, proteins, vectors etc.)**

| cfDNA extraction kit | QIAamp Circulating Nucleic Acid Kit (Qiagen, 55114) + QIAvac 24 Plus vacuum manifold |  |
| --- | --- | --- |
| cfDNA quantitation kit | Qubit dsDNA HS Assay kit + Qubit 2.0 Fluorometer |  |
| ddPCR | Bio-Rad’s QX200TM Digital Droplet PCR system | Bio-Rad Automated Droplet Generator (AutoDG), C1000 Touch™ thermal cycler (Bio-Rad), Bio-Rad QX-200 droplet reader |

- 1. **Please provide the details of the corresponding methods author for the manuscript:**

| Chiara Braconi, chiara.braconi@glasgow.ac.uk |
| --- |

**2.0 Please confirm for randomised controlled trials all versions of the clinical protocol are included in the submission. These will be published online as supplementary information.**

| Not applicable |
| --- |

**Gene fragments**

These positive controls are in antisense. Across exon 3 and intron 4. The wildtype triplet is CGT, or in antisense ACG.

**ddPCR™ Mutation Assay: IDH1 p.R132C, Human, Homo sapiens**

**Wildtype**

CCCATGAGACATACAAAAAGGTAATGCCGCCTCGCTAGGTGAGCTACAGCTCGATTGTCACGTTAAGCTGGCCTAAATATACAGTTATACATATATGCATTTCTCAATTTCATACCTTGCTTAATGGGTGTAGATACCAAAAGATAAGAATAAAACACATACAAGTTGGAAATTTCTGGGCCATGAAAAAAAAAACATGCAAAATCACATTATTGCCAACATGACTTACTTGATCCCCATAAGCATGAC**G**ACCTATGATGATAGGTTTTACCCATCCACTCACAAGCCGGGGGATATTTTTGCAGATAATGGCTTCTCTGAAGACCGTGCCACCCAGAATATTTCGTATGGTGCCATTTGGTGATTTCCACATTTGTTTCAACTTGAACTCCTCAACCCTCTTCTCATCAGGAGTGATAGTGGCACATTTGACGCCAACATTATGCTTCTTTATAGCTTCTGCAGCATC**CTG**GTCTCGACTATACGCCCGTTTTCGGATC

**Mutant**

CCCATGAGACATACAAAAAGGTAATGCCGCCTCGCTAGGTGAGCTACAGCTCGATTGTCACGTTAAGCTGGCCTAAATATACAGTTATACATATATGCATTTCTCAATTTCATACCTTGCTTAATGGGTGTAGATACCAAAAGATAAGAATAAAACACATACAAGTTGGAAATTTCTGGGCCATGAAAAAAAAAACATGCAAAATCACATTATTGCCAACATGACTTACTTGATCCCCATAAGCATGAC**A**ACCTATGATGATAGGTTTTACCCATCCACTCACAAGCCGGGGGATATTTTTGCAGATAATGGCTTCTCTGAAGACCGTGCCACCCAGAATATTTCGTATGGTGCCATTTGGTGATTTCCACATTTGTTTCAACTTGAACTCCTCAACCCTCTTCTCATCAGGAGTGATAGTGGCACATTTGACGCCAACATTATGCTTCTTTATAGCTTCTGCAGCATCCTGGTCTCGACTATACGCCCGTTTTCGGATC

c.394C>T

p.R132C (**C**GT>**T**GT)

**ddPCR™ Mutation Assay: IDH1 p.R132L, Human, Homo sapiens**

**Wildtype**

CCCATGAGACATACAAAAAGGTAATGCCGCCTCGCTAGGTGAGCTACAGCTCGATTGTCACGTTAAGCTGGCC**G**TAAATATACAGTTATACATATATGCATTTCTCAATTTCATACCTTGCTTAATGGGTGTAGATACCAAAAGATAAGAATAAAACACATACAAGTTGGAAATTTCTGGGCCATGAAAAAAAAAACATGCAAAATCACATTATTGCCAACATGACTTACTTGATCCCCATAAGCATGA**C**GACCTATGATGATAGGTTTTACCCATCCACTCACAAGCCGGGGGATATTTTTGCAGATAATGGCTTCTCTGAAGACCGTGCCACCCAGAATATTTCGTATGGTGCCATTTGGTGATTTCCACATTTGTTTCAACTTGAACTCCTCAACCCTCTTCTCATCAGGAGTGATAGTGGCACATTTGACGCCAACATTATGCTTCTTTATAGCTTCTGCAGCATC**CG**GTCTCGACTATACGCCCGTTTTCGGATC

**Mutant**

CCCATGAGACATACAAAAAGGTAATGCCGCCTCGCTAGGTGAGCTACAGCTCGATTGTCACGTTAAGCTGGCC**G**TAAATATACAGTTATACATATATGCATTTCTCAATTTCATACCTTGCTTAATGGGTGTAGATACCAAAAGATAAGAATAAAACACATACAAGTTGGAAATTTCTGGGCCATGAAAAAAAAAACATGCAAAATCACATTATTGCCAACATGACTTACTTGATCCCCATAAGCATGA**A**GACCTATGATGATAGGTTTTACCCATCCACTCACAAGCCGGGGGATATTTTTGCAGATAATGGCTTCTCTGAAGACCGTGCCACCCAGAATATTTCGTATGGTGCCATTTGGTGATTTCCACATTTGTTTCAACTTGAACTCCTCAACCCTCTTCTCATCAGGAGTGATAGTGGCACATTTGACGCCAACATTATGCTTCTTTATAGCTTCTGCAGCATCCGGTCTCGACTATACGCCCGTTTTCGGATC

c.395G>T

p.R132L (C**G**T>C**T**T)

**ddPCR™ Mutation Assay: IDH1 p.R132G, Human, Homo sapiens**

**Wildtype**

CCCATGAGACATACAAAAAGGTAATGCCGCCTCGCTAGGTGAGCTACAGCTCGATTGTCACGTTAAGCTGGCCTAAATATACAGTTATACATATATGCATTTCTCAATTTCATACCTTGCTTAATGGGTGTAGATACCAAAAGATAAGAATAAAACACATACAAGTTGGAAATTTCTGGGCCATGAAAAAAAAAACATGCAAAATCACATTATTGCCAACATGACTTACTTGATCCCCATAAGCATGAC**G**ACCTATGATGATAGGTTTTACCCATCCACTCACAAGCCGGGGGATATTTTTGCAGATAATGGCTTCTCTGAAGACCGTGCCACCCAGAATATTTCGTATGGTGCCATTTGGTGATTTCCACATTTGTTTCAACTTGAACTCCTCAACCCTCTTCTCATCAGGAGTGATAGTGGCACATTTGACGCCAACATTATGCTTCTTTATAGCTTCTGCAGCATCCTGGTCTCGACTATACGCCCGTTTTCGGATC

**Mutant**

CCCATGAGACATACAAAAAGGTAATGCCGCCTCGCTAGGTGAGCTACAGCTCGATTGTCACGTTAAGCTGGCCTAAATATACAGTTATACATATATGCATTTCTCAATTTCATACCTTGCTTAATGGGTGTAGATACCAAAAGATAAGAATAAAACACATACAAGTTGGAAATTTCTGGGCCATGAAAAAAAAAACATGCAAAATCACATTATTGCCAACATGACTTACTTGATCCCCATAAGCATGAC**C**ACCTATGATGATAGGTTTTACCCATCCACTCACAAGCCGGGGGATATTTTTGCAGATAATGGCTTCTCTGAAGACCGTGCCACCCAGAATATTTCGTATGGTGCCATTTGGTGATTTCCACATTTGTTTCAACTTGAACTCCTCAACCCTCTTCTCATCAGGAGTGATAGTGGCACATTTGACGCCAACATTATGCTTCTTTATAGCTTCTGCAGCATCCTGGTCTCGACTATACGCCCGTTTTCGGATC

c.394C>G

p.R132G (**C**GT>**G**GT)

**ddPCR™ Mutation Assay: IDH1 p.R132S c.394C>A, Human, Homo sapiens**

**Wildtype**

CCCATGAGACATACAAAAAGGTAATGCCGCCTCGCTAGGTGAGCTACAGCTCGATTGTCACGTTAAGCTGGCCTAAATATACAGTTATACATATATGCATTTCTCAATTTCATACCTTGCTTAATGGGTGTAGATACCAAAAGATAAGAATAAAACACATACAAGTTGGAAATTTCTGGGCCATGAAAAAAAAAACATGCAAAATCACATTATTGCCAACATGACTTACTTGATCCCCATAAGCATGAC**G**ACCTATGATGATAGGTTTTACCCATCCACTCACAAGCCGGGGGATATTTTTGCAGATAATGGCTTCTCTGAAGACCGTGCCACCCAGAATATTTCGTATGGTGCCATTTGGTGATTTCCACATTTGTTTCAACTTGAACTCCTCAACCCTCTTCTCATCAGGAGTGATAGTGGCACATTTGACGCCAACATTATGCTTCTTTATAGCTTCTGCAGCATCCTGGTCTCGACTATACGCCCGTTTTCGGATC

**Mutant**

CCCATGAGACATACAAAAAGGTAATGCCGCCTCGCTAGGTGAGCTACAGCTCGATTGTCACGTTAAGCTGGCCTAAATATACAGTTATACATATATGCATTTCTCAATTTCATACCTTGCTTAATGGGTGTAGATACCAAAAGATAAGAATAAAACACATACAAGTTGGAAATTTCTGGGCCATGAAAAAAAAAACATGCAAAATCACATTATTGCCAACATGACTTACTTGATCCCCATAAGCATGAC**T**ACCTATGATGATAGGTTTTACCCATCCACTCACAAGCCGGGGGATATTTTTGCAGATAATGGCTTCTCTGAAGACCGTGCCACCCAGAATATTTCGTATGGTGCCATTTGGTGATTTCCACATTTGTTTCAACTTGAACTCCTCAACCCTCTTCTCATCAGGAGTGATAGTGGCACATTTGACGCCAACATTATGCTTCTTTATAGCTTCTGCAGCATCCTGGTCTCGACTATACGCCCGTTTTCGGATC

c.394C>A

p.R132S (**C**GT>**A**GT)
