# Supplementary material for: IDH1 R132 mutations or HER2-positivity and benefit from platinum-based therapy for biliary tract cancers
Source: JHEP Rep. 2026 May 20;8(8):101899. doi: 10.1016/j.jhepr.2026.101899 (PMC13380706; doi:10.1016/j.jhepr.2026.101899)
Supplement: Multimedia component 5 [file mmc5.pdf]

IDH1 R132 mutations or HER2-positivity and benefit from platinum-based therapy for biliary tract cancers

Authors

Giulia Tesini, Jack Greaves, Olivia Knight, …, Alan Christie, Timothy J. Kendall, Chiara Braconi

Correspondence

chiara.braconi@glasgow.ac.uk (C. Braconi).

Graphical abstract

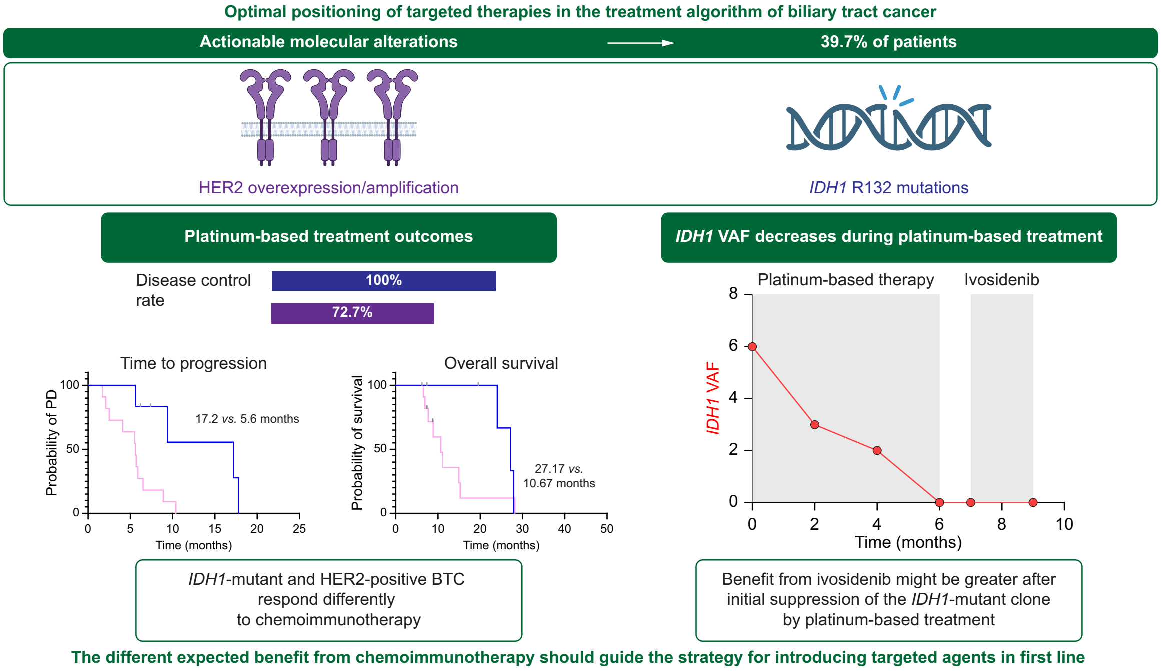

Highlights:

- Patients with *IDH1*-mutant and HER2-positive biliary tract cancer respond differently to chemoimmunotherapy.
- Platinum chemotherapy controls *IDH1*-mutated clones on longitudinal liquid biopsy.
- Expected benefit from chemoimmunotherapy should guide introduction of targeted agents in first line.

Impact and implications:

We conducted an analysis to determine whether patients with biliary tract cancer harboring the most common actionable alterations (*i.e.* *IDH1* R132 mutations and HER2 overexpression or amplification) had different patterns of response to first-line platinum-based chemo(immuno)therapy. The goal was to determine whether the introduction of targeted therapies in the first-line setting should be tailored based on the alteration detected. In our cohort, patients with *IDH1* R132 mutations experienced durable benefit from platinum-based chemotherapy, while those with HER2-positive tumors only experienced a short-lived benefit from it. Although these findings are exploratory and limited by small sample size, they may inform future strategies for earlier integration of targeted therapies and support the design of prospective clinical trials.

https://doi.org/10.1016/j.jhepr.2026.101899
© 2026 The Authors. Published by Elsevier B.V. on behalf of European Association for the Study of the Liver (EASL). This is an open access article under the CC BY license (http://creativecommons.org/licenses/by/4.0/).

IDH1 R132 mutations or HER2-positivity and benefit from platinum-based therapy for biliary tract cancers

Giulia Tesini[1,2], Jack Greaves[1,3], Olivia Knight[4], Holly Shand[5], Angela Ammirabile[1,2,6], Sophie McHaffie[7], Jen Milne[8], Halima Ibrahim[8], Rosemary Meenan[8], Sophia Campbell[8], Margaret Henderson[8], Andrea Lampis[9], Louis Chesler[9], Andrea Casadei-Gardini[10], Lorenza Rimassa[2,11], Alan Christie[3,12], Timothy J. Kendall[3,7,13], Chiara Braconi[1,3,8,*]

JHEP Reports **2026**. vol. 8 | 1–11

Check for updates

Background & Aims: Targeted therapies for biliary tract cancers (BTC) are approved in the second-line setting. We investigated response to first-line platinum-based palliative systemic anti-cancer treatment in patients with the most common druggable alterations to determine the optimal positioning of targeted therapies in the treatment algorithm.

Methods: Patients treated at the Beatson West of Scotland centralized BTC clinic who underwent genomic profiling were retrospectively selected; those with the most common actionable alterations who received platinum-based treatment were included in clinical analyses. Observations were supported by tracking *IDH1* mutations with digital droplet PCR on longitudinal cell-free DNA samples.

Results: Druggable alterations were identified in 39.7% of patients; HER2 overexpression/amplification and *IDH1* R132 mutations were the most commonly identified alterations. Compared to patients with HER2-positive tumors (n = 11), patients with *IDH1* R132 mutations (n = 6) had longer median time to best response (5.99 *vs*. 2.5 months; hazard ratio [HR] 0.28; 95% CI 0.09-0.91; $p = 0.0033$), and median time to progression (17.2 *vs*. 5.6 months; HR 0.266; 95% CI 0.0955-0.741; $p = 0.0075$). Compared to a cohort of patients without either alteration, those with *IDH1*-mutated BTC had longer median overall survival (HR 0.30; 95% CI 0.14-0.67; $p = 0.0229$); those with HER2-positive BTC showed a worse median time to progression (HR 1.97; 95% CI 0.85-4.58; $p = 0.0408$) and a trend towards worse median overall survival (HR 1.38; 95% CI 0.61-3.13; $p = 0.3884$). *IDH1* variant allele frequency decreased during first-line treatment, regardless of response; prolonged benefit from ivosidenib in the second line was observed when variant allele frequency was <1 at the start of targeted treatment.

Conclusions: These preliminary data suggest that targeted therapies may need to be introduced earlier, including in the first-line setting, with strategies tailored to specific molecular alterations. Access to platinum-based palliative systemic anti-cancer treatment remains particularly important for patients with *IDH1*-mutated BTC.

© 2026 The Authors. Published by Elsevier B.V. on behalf of European Association for the Study of the Liver (EASL). This is an open access article under the CC BY license (http://creativecommons.org/licenses/by/4.0/).

Introduction

Biliary tract cancers (BTCs), albeit rare in comparison to other tumors, have been increasing in incidence worldwide and are affecting a progressively younger demographic.[1,2] Curability rates are low, as only approximately 30% of patients present with resectable disease, and even when surgery is radical, the risk of relapse is 50%.[2–4] Prognosis for advanced disease is poor, with a median overall survival (OS) of 12 months in molecularly unselected populations with the newly introduced chemoimmunotherapy regimens of cisplatin, gemcitabine, and durvalumab or pembrolizumab.[5–8] Recently, precision medicine has reshaped the treatment algorithm for patients with BTC. Indeed, these tumors have a diverse molecular landscape: actionable alterations are identified in up to 50% of the cases and are enriched in intrahepatic cholangiocarcinoma (iCCA).[9] The most common actionable alterations are *IDH1* R132 mutations and *FGFR2* fusions, found in 15% and 10% of iCCA cases, respectively, and HER2 overexpression/amplification, documented in up to 15-20% of cases of extrahepatic CCA (eCCA) and gallbladder cancer (GBC).[9]

Current guidelines recommend that first-line palliative systemic anti-cancer treatment (pSACT) is offered with an all-comers approach, while targeted drugs are indicated only in second line and beyond.[10,11] However, their optimal collocation in the treatment algorithm for BTC is still under investigation. As targeted therapies have demonstrated unprecedented survival outcomes, with median OS exceeding 1 year in most cases in the second-line palliative setting, efforts are underway to move them into the first-line setting. This includes evaluation as single agents, in combination with chemotherapy and immune checkpoint inhibitors, or as maintenance therapy following a course of chemoimmunotherapy, as in the ABC-10 trial.[9,12]

We conducted a single-center retrospective analysis aimed at identifying a possible association between selected actionable molecular alterations and response to first-line

* Corresponding author. Address: School of Cancer Sciences, University of Glasgow, Glasgow, G61 1QH, UK. Tel.: +44(0) 141 330 3278; fax: +0141 942 6521.
E-mail address: chiara.braconi@glasgow.ac.uk (C. Braconi).
https://doi.org/10.1016/j.jhepr.2026.101899

platinum-based pSACT. We leveraged the fact that all patients with BTC in the West of Scotland are followed at the centralized West of Scotland BTC clinic, guaranteeing a homogenous management of cases, with full access to longitudinal clinical annotations and RECIST review of radiological assessments. We focused on *IDH1* R132 mutations and HER2 overexpression/amplifications not only because of their high prevalence, but also because studies with targeted drugs in the first-line setting are ongoing.[9,13–15] The goal was to establish whether different strategies need to be adopted when incorporating anti-IDH1 and anti-HER2 agents in the first-line setting, based on the clinical behavior exhibited in response to platinum-based chemotherapy.

Materials and methods

Population

All patients who underwent tissue genomic profiling between June 2022 (when the GENTLE molecular profiling program was instituted) and September 30, 2025, were retrospectively selected. To better reflect our clinic population, patients who underwent panel-based next-generation sequencing either on tissue through private, out-of-pocket testing (n = 2) or on liquid biopsy specimens (n = 4) were also included (Fig. 1A). All patients were enrolled in the REG-Bil translational study (IRAS: 276732; REC 20/SW/0054; ISRCTN: 15141439); one additional patient who underwent liquid biopsy, received first-line pSACT

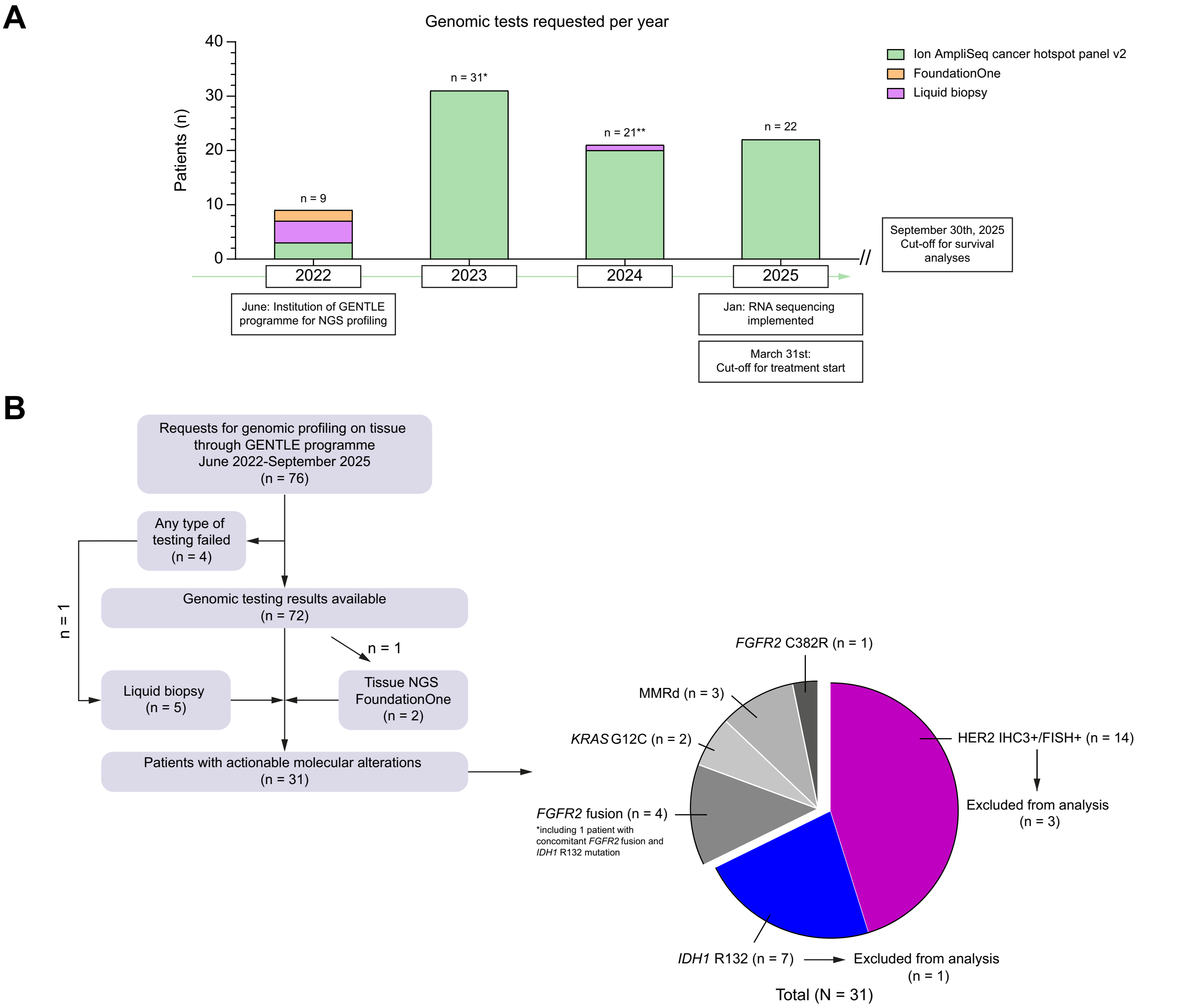

Fig. 1. Description of study population. (A) Summary of genomic tests requested per year from 2022 until September 2025. (B) Diagram of patients included in the study. Only patients with *IDH1* R132 mutations and HER2 positivity were retained for analyses; four patients with these alterations were excluded (received <1 cycle of pSACT, n = 2; started pSACT after March 31st, 2025, n = 1; resected and never relapsed, n = 1). IHC, immunohistochemistry; MMRd, mismatch repair deficiency; pSACT, palliative systemic anti-cancer treatment.

in Edinburgh, and was later referred to our center to receive second-line targeted treatment as part of a clinical trial was also included. All patients signed an informed consent form.

Molecular analyses on tissue

IDH1 R132, *KRAS* G12C, and *FGFR2* mutations at baseline were identified through DNA sequencing. Panels utilized for genomic analyses on tissue were the Ion AmpliSeq Cancer Hotspot v2 (n = 76, all in the GENTLE programme) and FoundationOne (n = 2), while panels utilized for liquid biopsy were the Avenio ctDNA Expanded kit v2 (n = 3) and FoundationOne (n = 2) (Tables S1–3). For patients included in the GENTLE program, *FGFR2* fusions were assessed using fluorescence *in situ* hybridization (FISH); from January 2025 onward, RNA sequencing was also implemented, investigating *FGFR2, NTRK1-3*, and *RET* fusions with the Oncomine Precision Assay GX, with reporting restricted to fusions and rearrangements in these three genes. For all other patients, *FGFR2* fusions, when investigated, were tested through RNA sequencing as part of the FoundationOne panel (Table S2). HER2-positive status was defined either as an immunohistochemistry (IHC) score of 3+ (using the criteria utilized in gastric cancer), or as a FISH with HER2/neu:CEP17 ratio ≥2, as recently published data point to a concordance of 94% between the two techniques.[16,17] Mismatch repair deficiency was determined using IHC.

Clinical analyses

After identifying the most common actionable alterations in our cohort, an association with response to platinum-based chemotherapy was investigated. All patients with these alterations who received at least one cycle of first-line platinum-based pSACT were selected. Date of data cut-off for survival analyses was September 30th, 2025. March 31st, 2025 was chosen as the cut-off for start of first-line pSACT in order to have at least a 6-month-long follow-up for each patient, corresponding to eight cycles of chemotherapy +/- immunotherapy (Fig. 1B). A reference cohort was also identified, including all patients who underwent genomic profiling without the molecular alterations of interest and meeting the aforementioned inclusion criteria (Fig. S1).

Response to treatment was assessed through CT scans every 3 months as per local clinical practice guidelines, unless otherwise indicated. Objective response rate was defined as the proportion of patients who had a complete response (CR) or partial response (PR) to treatment, while disease control rate (DCR) was defined as the proportion of patients who achieved CR, PR, or stable disease (SD). Time to best response (BR) was calculated from treatment start to maximum radiological response (CR, PR or SD). Duration of clinical benefit was calculated from time of first confirmed radiological benefit (CR, PR or SD) to progressive disease (PD) or censored at date of last follow-up for patients who had not progressed yet. CT scans of patients with actionable alterations of interest were reviewed by two dedicated radiologists (OK and AA) to determine response according to RECIST v1.1 and inform the optimal integration of targeted therapies into the treatment algorithm. Time to progression (TTP) and OS were calculated from treatment initiation to PD and to death or last follow-up, respectively, using the Kaplan–Meier method. PFS1/PFS2 was defined as the ratio between progression-free survival (PFS) to first (PFS1) and second-line (PFS2) therapy, with PFS being calculated from start of treatment until PD or death from any cause with the Kaplan-Meier method. Continuous variables were compared using Mann-Whitney *U* test, whilst categorical variables were compared using Fisher's exact test. Statistical significance was accepted for p values <0.05. Statistical analyses were conducted using GraphPad Prism (version 10.5.0).

Longitudinal analysis of cell-free DNA through digital droplet PCR

Clinical observations were supported through translational analysis, tracking *IDH1* R132 mutations with digital droplet PCR (ddPCR) on cell-free DNA (cfDNA) extracted from plasma samples collected during treatment in a longitudinal fashion.

Plasma samples from patients were collected within 4 h from blood collection. Briefly, blood samples were centrifuged at 800 x g at room temperature for 10 min with brake off; plasma was carefully collected and stored at -80 °C for future use*.* Subsequently, cfDNA was extracted using the QIAamp Circulating Nucleic Acid Kit (Qiagen, 55114) on the QIAvac 24 Plus vacuum manifold, following the manufacturer's protocol. Plasma samples were gently thawed and centrifuged (5 min, 16,000 x *g*, 4 °C) to remove cryoprecipitates. Samples were incubated with Proteinase K to degrade contaminant proteins. Carrier RNA (1 µg per sample) was added to enhance subsequent binding to the silica membrane. Following appropriate washes with the vacuum manifold, cfDNA was eluted into buffer AVE and quantified using the Qubit dsDNA HS Assay kit on a Qubit 2.0 Fluorometer, following the manufacturer's protocol. Samples were subsequently stored at -20 °C for future use for ddPCR.

ddPCR was conducted using Bio-Rad's QX200^{TM} Digital Droplet PCR system. cfDNA samples were mixed with ddPCR Supermix for Probes (No dUTP) and mutation-specific ddPCR Mutation Detection Assays probes. Samples were then partitioned into droplets using the Bio-Rad Automated Droplet Generator (AutoDG) using Automated Droplet Generation Oil for Probes. PCR amplification of samples was carried out on a C1000 Touch^{TM} thermal cycler (Bio-Rad) under the following conditions: 95 °C for 10 min, 40 cycles of 94 °C for 30 s and 55 °C for 60 s (optimum annealing temperature per BioRad validated probes, temperature increment was +2 °C/s), 98 °C for 10 min. Droplets were read on the Bio-Rad QX-200 droplet reader and data analysis was performed using QuantaSoft v2.2.

Results

Between June 2022 and September 2025, 83 molecular profiling analyses were requested, with two patients having genomic testing both on tissue and on liquid biopsy (Fig. 1A). Genomic profiling was requested in patients with advanced disease who were eligible for first-line pSACT in 94% of cases (platinum-based pSACT, n = 75; gemcitabine, n = 1), according to European Society for Medical Oncology (ESMO) guidelines.[10,11] In addition, four patients underwent genomic profiling after curative surgery and one after a locoregional procedure as part of a research program; these five patients did not receive pSACT (Fig. 1A). In four cases (4.76%; 3

perihilar CCA, 1 GBC), testing on tissue failed due to low cellularity (<0.67 ng/µl of DNA with <10% neoplastic cell content), and repeat testing on liquid biopsy was performed in one of these patients (Fig. 1B).

Tissue was sufficient for testing at least one alteration in 78 cases (96.3%): 39 (50%) had iCCA, while the remaining patients were equally distributed across other BTC subtypes (Fig. 2A). Thirteen patients (15.6%) underwent only limited gene panel analysis due to insufficient tissue for extended profiling (Fig. 2A). Among 65 patients who underwent DNA sequencing, the most frequently altered genes were *TP53* (23 patients, 35.4%) and *KRAS* (20 patients, 30.8%), with the G12D variant accounting for 50% of *KRAS* mutations (Fig. S2A). No differences in the distribution of *TP53* or *KRAS* alterations were observed across BTC subtypes (Fig. S2B and C).

Actionable molecular alterations were documented in 31 cases (39.7%). The most common targetable alteration was HER2 overexpression (3+ IHC) or amplification (FISH positive), which was identified in 14 patients (17.9% of the whole cohort,

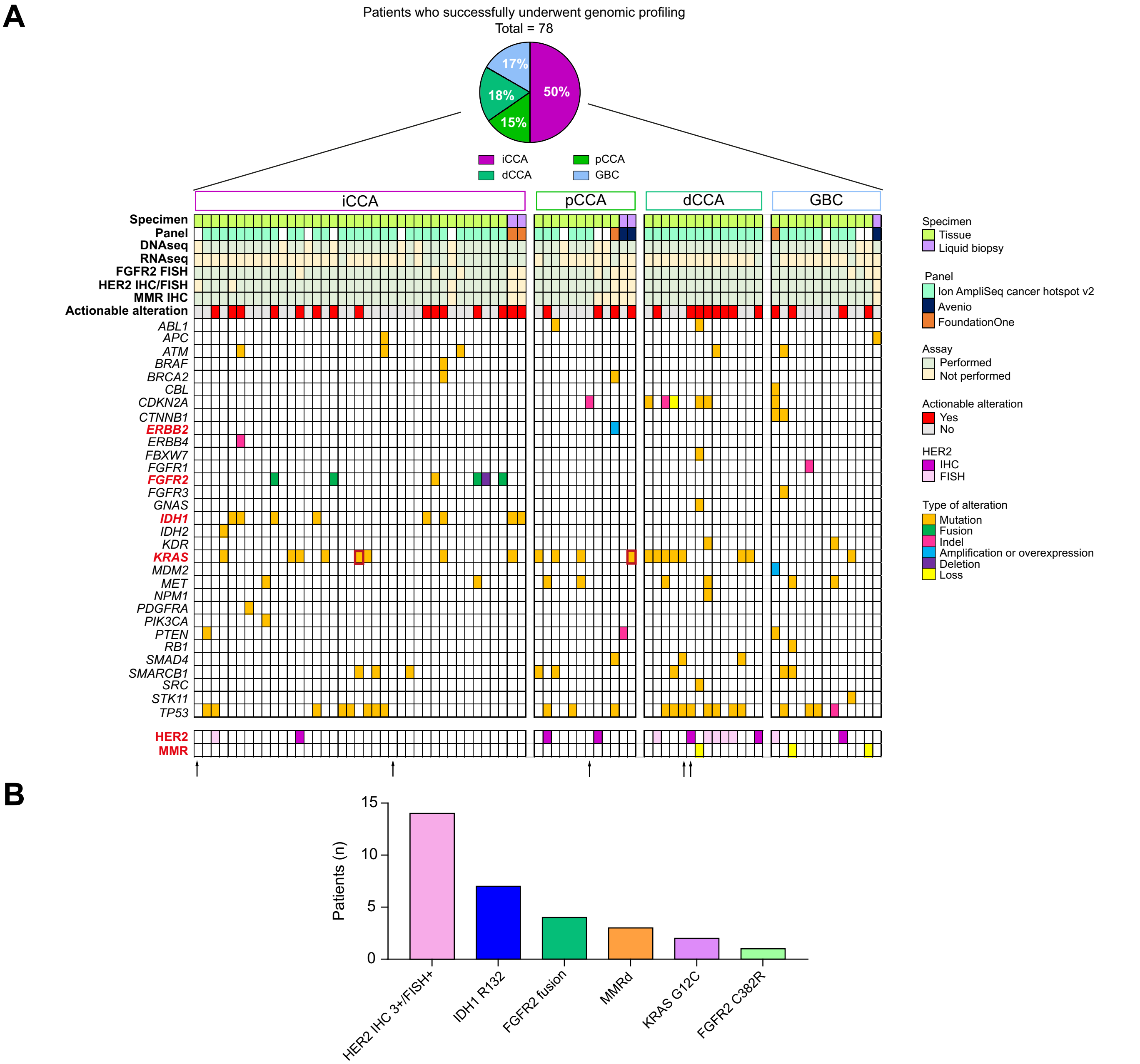

Fig. 2. Molecular alterations identified in the population who underwent molecular profiling. (A) Genes of interest are highlighted in red. If no panel is indicated, tissue was sufficient only for single-gene analysis. Among *KRAS* mutations, squares with red borders indicate the G12C mutation. Arrows indicate patients who underwent surgery and never received palliative treatment. MMR deficiency was determined via IHC for PMS2, MSH2, MSH6, and MLH1. (B) Summary of actionable alterations identified. dCCA, distal cholangiocarcinoma; FISH, fluorescence *in situ* hybridization; GBC, gallbladder cancer; iCCA, intrahepatic cholangiocarcinoma; IHC, immunohistochemistry; indel, insertion-deletion; MMR, mismatch repair; pCCA, perihilar cholangiocarcinoma.

20.3% of those who underwent dedicated testing): six patients had an IHC score of 3+ and eight had a positive FISH, predominantly with eCCA or GBC (p = 0.006) (Fig. S2D). In the overall cohort, *IDH1* R132 mutations were documented in eight patients (10.3%), all with iCCA; *FGFR2* fusions were identified in four patients (5%), and one of them had a concomitant *IDH1* R132 mutation; mismatch repair deficiency was identified in three patients (3.8%), and *KRAS* G12C mutations in two (2.6%). One patient had an *FGFR2* C382R mutation, which has been reported to be sensitive to pemigatinib (Figs 1B and 2B).[18–20]

To determine if any actionable molecular alteration was associated with response to chemotherapy, we focused on the subgroup of patients who underwent platinum-based pSACT. As HER2 overexpression/amplifications and *IDH1* R132 mutations were the most represented druggable alterations in our cohort and clinical trials studying these targeted agents in the first-line setting are ongoing,[13–15] we focused our analyses on these two subgroups. We aimed to understand if the introduction of anti-HER2 and anti-IDH1 drugs in the first-line setting needs to follow different strategies and to provide insights for the interpretation of results from ongoing studies.

We identified 11 patients with HER2-positive BTC (4 IHC-positive/7 FISH-positive) and six with an *IDH1* R132 mutation meeting the inclusion criteria (Fig. 1B). Baseline characteristics of this population are summarized in Table 1. No statistically significant differences were identified between the two subgroups, except for primary tumor site, as previously described. At the time of data cut-off, all patients with HER2-positive tumors and 67% of those with an *IDH1* mutation had progressed, whilst 81% and 50% of them had died, respectively. A reference cohort of 40 patients was identified (Fig. S1, Table S4); at the time of data cut-off, 83% had progressed and 73% had died.

ORR was 67% among patients with *IDH1*-mutated BTC compared to 36% among those with HER2-positive tumors, but the difference was not statistically significant, possibly due to the small sample size (p = 0.3348) (Fig. 3A). Notably, all patients with an *IDH1* R132 mutation achieved disease control, while DCR was 72.7% among those with HER2-positive BTC (p = 0.5147) (Fig. 3B); by comparison, the DCR in our reference cohort was 75%.

As shown in the waterfall plot (Fig. 3C), patients with *IDH1*-mutated BTC were the ones who benefitted the most from pSACT in terms of tumor shrinkage, regardless of access to immunotherapy. Interestingly, among patients who achieved disease control, all patients with a HER2-positive tumor had their BR at the first CT scan assessment (median time to BR: 2.76 months; IQR, 2.28-3.03), whilst 83% of patients with *IDH1*-mutated BTC had maximum radiological response after >5 months from treatment start, yielding a median time to BR of 5.99 months (IQR, 4.99-8.39) (hazard ratio [HR] 0.28; 95% CI 0.09-0.91; p = 0.0033) (Fig. 3D, Fig. S3). Median duration of clinical benefit was also numerically longer in the *IDH1*-mutated group than in the HER2-positive group (9.15 months [95% CI 2.9-+infinity] *vs*. 3.05 months [95% CI 2.4-5.8]; p = 0.0586) (Fig. 3E).

Patients with *IDH1*-mutated BTC had longer median TTP than those with HER2-positive BTC (17.2 months *vs*. 5.6 months; HR 0.266; 95% CI 0.0955-0.741; p = 0.0075). In particular, 50% of patients with *IDH1* R132 mutations had a TTP >8 months; among the other half, two out of three are still on treatment with maintenance immunotherapy, both having received one administration before data cut-off. Among patients with HER2-positive tumors, only 2 (18%) had a TTP >8 months, while 28% were primary refractory to chemo(-immuno)therapy (Fig. 3C,F,G). There was also a trend towards a better median OS among patients with *IDH1* R132 mutations (27.17 *vs.* 10.67 months; HR 0.397; 95% CI 0.127-1.24; p = 0.1187) (Fig. 3H). In comparison to our reference cohort, patients with *IDH1*-mutated tumors showed a trend towards longer median TTP (HR 0.54; 95% CI 0.23-1.24; p = 0.2214) and significantly longer median OS (HR 0.30; 95% CI 0.14-0.67; p = 0.0229). By contrast, those with HER2-positive BTC had shorter median TTP (HR 1.97; 95% CI 0.85-4.58; p = 0.0408) and median OS (HR 1.38; 95% CI 0.61-3.13; p = 0.3884), but the OS difference was not statistically significant (Fig. 3G,H).

All patients with an *IDH1* R132 mutation who progressed to first-line pSACT received second-line ivosidenib. Among patients with HER2-positive BTC, 2 (18%) did not receive any targeted therapy (one due to clinical deterioration and one due to lack of access to anti-HER2 drugs), whilst the others received either trastuzumab deruxtecan (n = 5, 45%) or

Table 1. Baseline characteristics of population of interest.

	IDH1 R132 (n = 6)	HER2-positive (n = 11)	*p* value
Female, n(%)	5 (83)	6 (55)	0.3334
Median age at diagnosis, years (IQR)	59 (43-69)	65 (57-72)	0.8075
BTC subtype, n(%)			
iCCA	6 (100)	2 (18)	0.0023
pCCA	0	1 (9)	
dCCA	0	6 (55)	
GBC	0	2 (18)	
Resectable disease at diagnosis, n (%)	0	2 (18)	0.5147
1L pSACT, n (%)			
Platinum/gemcitabine/durvalumab	3 (50)	8 (73)	0.6
Platinum/gemcitabine	3 (50)	3 (27)	
Median CA19-9 at start of 1L pSACT, kU/L (IQR)*	48 (26-912.5)	199 (45-3,585)	0.4409
Median CEA at start of 1L pSACT, µg/L (IQR)*	6.1 (3-14.5)	7.3 (2.2-86.6)	0.3773

Continuous variables were compared using Mann-Whitney *U* test, categorical variables using Fisher's exact test.
1L, first line; BTC, biliary tract cancer; CA19-9, carbohydrate antigen 19-9; CEA, carcinoembryonic antigen; dCCA, distal cholangiocarcinoma; GBC, gallbladder cancer; iCCA, intrahepatic cholangiocarcinoma; IQR, interquartile range; pCCA, perihilar cholangiocarcinoma; pSACT, palliative systemic anti-cancer treatment.
*Data missing for one patient with *IDH1*-mutated BTC.

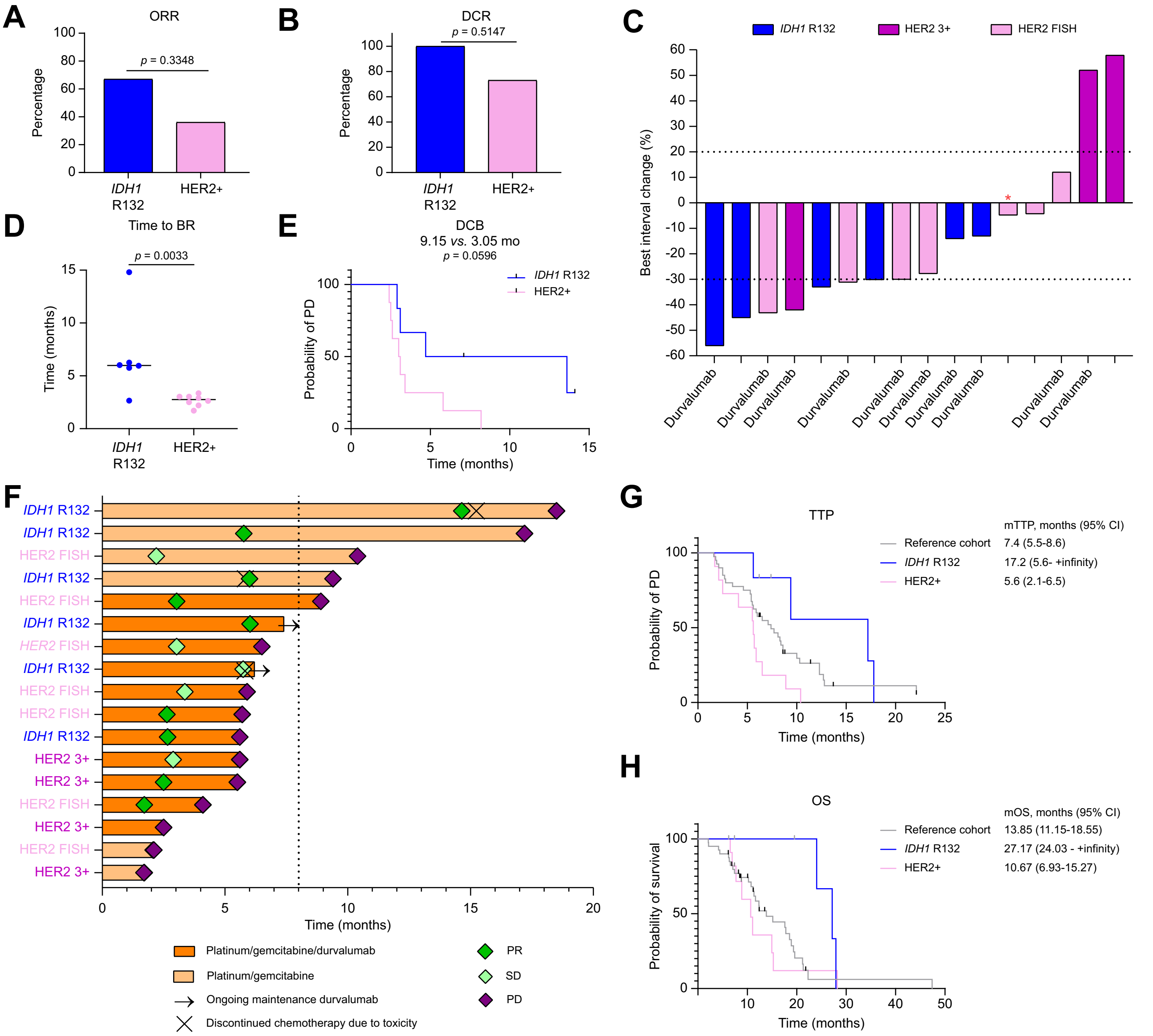

Fig. 3. Outcomes with first-line platinum-based pSACT among patients with *IDH1* R132 mutations and HER2 overexpression/amplification. (A) ORR (Fisher's exact test); (B) DCR (Fisher's exact test) and (C) waterfall plot. The red asterisk indicates a patient who had PD due to new-onset metastases despite a decrease in size of target lesions. For one patient with a HER2 IHC 3+ tumor, best % interval change per RECIST v1.1 could not be calculated, as baseline assessment was an FDG-PET, while following assessments were performed through CT scans; the patient achieved disease control after four cycles of chemoimmunotherapy but progressed after the 8th cycle. (D) Time to BR (line set at median; Kaplan-Meier). (E) DCB (Kaplan-Meier). (F) Swimmer plot; a reference line has been set at 8 months, corresponding to the expected median PFS with cisplatin, gemcitabine, and durvalumab.[5,6]. (G) TTP (Kaplan-Meier). (H) OS (Kaplan-Meier). BR, best response; DCB, duration of clinical benefit; DCR, disease control rate; FISH, fluorescence *in situ* hybridization; IHC, immunohistochemistry; ORR, objective response rate; OS, overall survival; PD, progressive disease; PR, partial response; SD, stable disease; TTP, time to progression.

FOLFOX and trastuzumab (n = 4, 36%). To determine the clinical benefit derived from pSACT compared to second-line targeted treatment, we calculated PFS1/PFS2 ratios; we considered exclusively patients who had already progressed to second line to have a complete follow-up. 75% of patients with HER2-positive tumors had a PFS1/PFS2 greater than 1 (range 0.74-3.63), while all patients with *IDH1* R132 mutations had a PFS1/PFS2 ratio >4 (range 4.5-6.2) (Fig. 4). This suggests that patients with IDH1-mutated BTC may derive greater benefit from platinum-based therapy, supporting the notion that chemotherapy should be considered in these cases to achieve optimal clinical outcomes. Only one patient in our cohort has achieved durable disease control with ivosidenib, has been on treatment for over 1 year, and currently has a PFS1/PFS2 ratio of <1 (0.4).

In light of these clinical observations, we conducted a longitudinal analysis of *IDH1* R132 mutations on liquid biopsy using ddPCR to assess the response of *IDH1*-mutated clones to pSACT. We observed a consistent downward trend in the variant allele frequency (VAF) of these alterations during

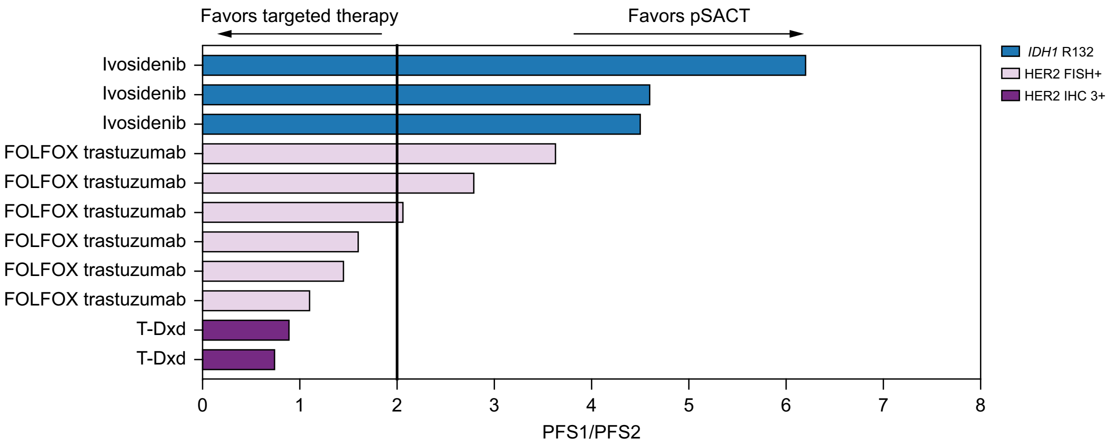

Fig. 4. PFS1/PFS2 ratio for patients who received second-line targeted therapy. A reference line has been set at 2, corresponding to the ratio between the expected median PFS to cisplatin, gemcitabine, and durvalumab (8 months) and the one to standard second-line FOLFOX (4 months).[5,6,21] Reference PFS1/PFS2 ratios are 4 for ivosidenib (expected median PFS: 2 months), 1.6 for FOLFOX+trastuzumab (expected median PFS: 5 months), 1.14 for T-Dxd (expected median PFS for IHC 3+ patients: 7 months).[22–25] IHC, immunohistochemistry; PFS, progression free survival; pSACT, palliative systemic anti-cancer treatment; T-Dxd, trastuzumab deruxtecan.

platinum-based pSACT, regardless of whether patients achieved PR or SD as BR (Fig. 5A-E). A subsequent increase in *IDH1* R132 VAF at PD was observed in patients who discontinued chemotherapy for more than 1 month before initiating second-line therapy (RB061, RB075) (Fig. 5A,B). This increase was not observed in the patient who commenced second-line ivosidenib within 1 month of PD on first-line pSACT (RB112) (Fig. 5C). Notably, this is also the only patient who experienced prolonged benefit from ivosidenib. Cooccurring molecular alterations did not seem to influence this pattern. The only patient who was primary refractory to platinum-based pSACT was the one with a concomitant *FGFR2* fusion: even in this case, the VAF of the *IDH1*-mutated clone decreased during first-line treatment and was <1 at PD (RB085) (Fig. 5F), seemingly supporting the notion that *IDH1*-mutated clones are chemo-sensitive and PD might be driven by other concomitant alterations.

Discussion

Our analysis needs to be contextualized within the developing landscape of targeted therapies for BTC. As targeted therapies in the second-line setting have improved clinical outcomes in pretreated patients,[9] their role in earlier treatment lines is worth exploring, and trials evaluating their efficacy in the first-line setting are ongoing.[13–15] In order to determine when it is best to introduce targeted therapies in the therapeutic algorithm, we set out to identify patterns of clinical behavior in response to platinum-based pSACT among patients with selected molecular alterations. We compared patients with HER2-positive and *IDH1*-mutated BTC to underscore the differences in clinical behavior between different types of molecularly altered BTC, to stress the importance of identifying tailored treatment strategies, and to highlight how choosing the timing of administration of targeted treatment should not follow a one-size-fits-all strategy.

We observed a short-lived benefit from platinum-based pSACT among patients with HER2 overexpression/amplification, with a median OS of 10.7 months and TTP of 5.6 months, which are lower than those expected with chemoimmunotherapy (12 and 8 months, respectively)[5–8] and shorter than those observed in our reference cohort. This is in line with emerging data that HER2 overexpression is a negative prognostic factor and that HER2 amplification is associated with more aggressive features, such as larger tumor size and more advanced stage at diagnosis.[26,27]

Of the three patients in our cohort who were primary refractory, two received chemotherapy alone, which is no longer the standard of care.[10,11] However, even among those who achieved disease control, maximum response was observed at the first CT scan assessment and benefit was promptly lost at the subsequent one in all but two patients: both of these patients had HER2 positivity determined by FISH; one received chemotherapy alone, while the other received chemotherapy in combination with durvalumab. It has recently been suggested that BTCs with a HER2 IHC score of 3+ and those that are HER2 2+/ISH+ may respond differently to chemoimmunotherapy, as the former exhibit an immune-desert tumor microenvironment (TME) in 70% of cases, whereas the latter present an inflamed TME in approximately one third of cases, and thus may have a higher likelihood of responding to immune checkpoint inhibitors.[28] Indeed, in an exploratory analysis of the TOPAZ-1 trial of cisplatin, gemcitabine, and durvalumab *vs.* chemotherapy alone, *ERBB2* amplifications were more frequent among long-term survivors (LTS) in the control arm (8.3% *vs.* 1.5%) and had a HR for OS and PFS >1, suggesting a lack of benefit from immunotherapy in this subgroup.[29,30] Even in our small cohort, preliminary signals suggested greater benefit from first-line pSACT in patients with HER2 FISH-positive tumors, whereas those with HER2 3+ IHC tumors had a TTP of <6 months and appeared to derive more prolonged benefit from targeted therapy in the second-line setting. With the limitation that definition of HER2-positive status differs across studies, the concordance of published data supports the rationale for trials assessing anti-HER2 agents in the first-line setting, either alone or in association with chemotherapy, especially for patients with HER2 3+ BTC.[13,15] Furthermore, the TME differences observed based on HER2 score on IHC highlight the importance of refining the

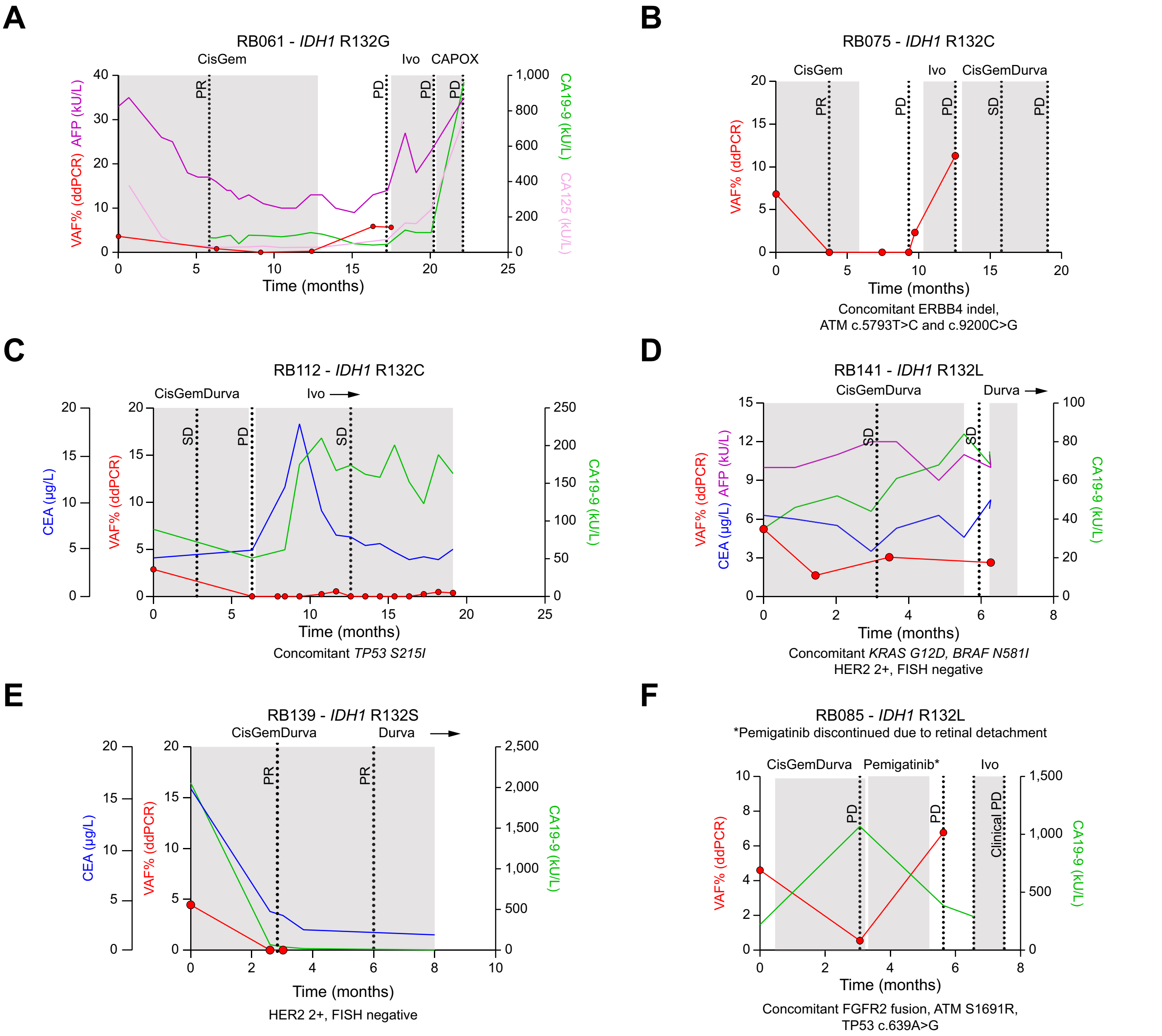

Fig. 5. Variation of *IDH1* R132 VAF as measured by ddPCR on cfDNA extracted by plasma samples collected longitudinally during treatment. VAF for *IDH1* R132 mutations decreases during platinum-based treatment, regardless of radiological response. Each panel (A-F) represents a different patient. Tumor markers are plotted only if elevated. AFP, alpha-fetoprotein; CA19-9, carbohydrate antigen 19-9; CEA, carcinoembryogenic antigen; CAPOX, capecitabine+oxaliplatin; CisGemDurva, cisplatin, gemcitabine, durvalumab; ddPCR, digital droplet PCR; IHC, immunohistochemistry; ivo, ivosidenib; PD, progressive disease; PR, partial response; SD, stable disease; VAF, variant allele frequency.

scoring method for HER2 in BTC, as it is possible that different levels of HER2 expression reflect different clinical behaviors and require diverse treatment approaches.

By contrast, we observed that patients with *IDH1* R132 mutations had durable clinical benefit from platinum-based pSACT, with a DCR of 100%, and longer median TTP and median OS than those observed in our reference cohort: this represents very preliminary evidence that access to chemotherapy is crucial for this subgroup. On a molecular level, we corroborated this data by observing a decrease in *IDH1* R132 VAF during platinum-based treatment, with a subsequent increase only after chemotherapy discontinuation. Based on these findings, it might be suggested that patients with *IDH1*-mutated BTC should have access to platinum-based chemotherapy in the first-line setting.

There is conflicting data on the role of *IDH1* mutations in mediating platinum-sensitivity. Preclinical data indicate that they may inhibit homologous recombination, a mechanism of DNA repair, via accumulation of the oncometabolite 2-hydroxyglutarate, suggesting a possible association with a BRCA-like phenotype.[31] Indeed, in other tumor types, *IDH1* mutations mediate sensitivity to PARP inhibitors, which is reversed when IDH1 inhibitors are administered.[32,33] These findings have been difficult to translate into clinical practice for

patients with BTC: data on platinum-sensitivity derive mostly from retrospective studies, where patients with *IDH1*-mutated BTC did not seem to have greater clinical benefit from chemotherapy compared to those with wild-type disease.[34,35] However, in an exploratory analysis of the TOPAZ-1 trial, *IDH1* mutations were more frequent among LTS (12% *vs.* 7.7%) and, particularly, in LTS treated with cisplatin, gemcitabine and durvalumab rather than chemotherapy alone (15% *vs.* 8.3%),[29] reinforcing the notion that standard-of-care chemo-immunotherapy is the first choice for *IDH1*-mutated patients.

Among our patients, the only one who experienced a prolonged benefit from ivosidenib had a suppressed *IDH1*-mutated clone (VAF <1) at the beginning of targeted treatment. All other patients who received ivosidenib were primary refractory: while cfDNA samples during targeted treatment were available only for one of them, it is noteworthy that *IDH1* VAF increased during targeted therapy. This might indicate that ivosidenib can keep the *IDH1*-mutated clone at bay but might not be as effective as chemotherapy at inducing its remission. It could be argued that platinum-based chemotherapy might induce resistance to ivosidenib. However, to our knowledge, there are no data to support this theory. Based on our results, we can only speculate that ivosidenib might not be a good candidate to replace cisplatin, gemcitabine, and durvalumab in the first line, and that the design of the ABC-10 trial, where targeted treatment is administered as maintenance therapy after 4 cycles of chemo-immunotherapy,[12] might be the most appropriate approach for patients with *IDH1* R132 mutations. However, in 67% of our patients, there was a continued improvement in radiological response during treatment, with maximum response being achieved at the second CT scan assessment or later. This might suggest that completing eight cycles of platinum-based pSACT as per standard of care before commencing maintenance ivosidenib might be worthwhile to maximize outcomes.

Our study has several limitations. Firstly, our cohort is small; therefore, the results need to be validated in larger prospective studies and our conclusions can only be considered hypothesis-generating. Besides, due to the limited study population, it is difficult to ascertain the role of confounding factors, such as anatomical BTC subtype, which is associated with these molecular alterations (*i.e. IDH1* mutations are found exclusively in iCCAs, while HER2 positivity is more common in GBCs/eCCAs) and has prognostic implications.[36,37] Moreover, the HER2-positive group is heterogeneous with regards to the methods used to define HER2 positivity, reflecting the evolving knowledge in this field: while the guidelines for HER2 scoring in gastric cancer are recommended,[11] a tumor-specific IHC score has not been validated yet. Besides, targeted treatments administered vary in this subgroup. The latest ESMO guidelines recommend trastuzumab deruxtecan or zanidatamab for patients with HER2 IHC 3+.[11] However, in previous years, limited access to these drugs in Scotland meant that patients were treated with a combination of FOLFOX and trastuzumab, which was originally assessed in a Korean-only study, thus limiting the generalizability of its results to Western populations.[23]

In conclusion, our analysis underscores that there might be differences in biological behavior in response to platinum-based pSACT between BTCs with HER2 overexpression/amplification and *IDH1* R132 mutations, identifying two subgroups of patients who might have different therapeutic needs. The former might benefit from first-line targeted therapies, potentially with distinct strategies based on HER2 expression levels, while – in the latter group – access to platinum-based pSACT might be crucial for therapeutic success and integration of ivosidenib as a maintenance strategy might be the optimal approach. Validation in larger prospective cohorts is mandatory.

Affiliations

[1]*School of Cancer Sciences, University of Glasgow, Glasgow, UK;* [2]*Department of Biomedical Sciences, Humanitas University, Via Rita Levi Montalcini, 4, 20072 Pieve Emanuele, Milan, Italy;* [3]*CRUK Scotland Centre, Switchback Rd, Glasgow, UK;* [4]*Department of Clinical Radiology, Glasgow Royal Infirmary, 84 Castle Street, Glasgow, UK;* [5]*School of Medicine, Dentistry and Nursing, University of Glasgow, Glasgow, UK;* [6]*Department of Diagnostic and Interventional Radiology, IRCCS Humanitas Research Hospital, via Manzoni 56, 20089 Rozzano, Milan, Italy;* [7]*Molecular Pathology, Department of Laboratory Medicine, Royal Infirmary of Edinburgh, 51 Little France Crescent, Old Dalkeith Road, Edinburgh, UK;* [8]*Beatson West of Scotland Cancer Centre, 1053 Great Western Rd, Glasgow, UK;* [9]*The Institute of Cancer Research, 15 Cotswold Rd, Sutton, UK;* [10]*Department of Oncology, Vita-Salute San Raffaele University, IRCCS San Raffaele Scientific Institute Hospital, Milan, Italy;* [11]*HepatoPancreatoBiliary Oncology Unit, Humanitas Cancer Center, IRCCS Humanitas Research Hospital, Via Alessandro Manzoni 56, 20089 Rozzano, Milan, Italy;* [12]*Edinburgh Cancer Centre, Western General Hospital, Edinburgh, UK;* [13]*Centre for Inflammation Research, Institute for Regeneration and Repair, University of Edinburgh, 5 Little France Drive, Edinburgh, UK*

Abbreviations

BR, best response; BTC, biliary tract cancer; cfDNA, cell-free DNA; CR, complete response; DCR, disease control rate; ddPCR, digital droplet polymerase chain reaction; eCCA, extrahepatic cholangiocarcinoma; FISH, fluorescence *in situ* hybridization; GBC, gallbladder cancer; HR, hazard ratio; iCCA, intrahepatic cholangiocarcinoma; IHC, immunohistochemistry; LTS, long-term survivors; OS, overall survival; PD, progressive disease; pSACT, palliative systemic anti-cancer treatment; PFS, progression-free survival; PFS1, progression-free survival on first-line therapy; PFS2, progression-free survival on second-line therapy; PR, partial response; SD, stable disease; TME, tumor microenvironment; TTP, time to progression; VAF, variant allele frequency.

Financial support

The REG-Bil study is sponsored by NHS Greater Glasgow and Clyde. GT has been supported by a Short Term Scientific Mission grant from the Precision-BTC-Network (COST Action 22125). The laboratory of CB has been supported by the Chief Scientist Office (TCS/21/25), University of Glasgow (Lord Kelvin Adam Smith Readership; Welcome Trust-Institutional ISSF Excellent and Catalyst award 31038), the CRUK-Scotland Centre (CTRQQR-2021\100006), AMMF (322067) Avacta (316813), Servier (320463) and Medannex (318434).

Conflicts of interest

GT received honoraria as speaker from AstraZeneca. SMH received honoraria as speaker and travelling fees from Servier Laboratories Ltd. ACG received consulting fees and honoraria as speaker from AstraZeneca, Bayer, BMS, Eisai, Incyte, Ipsen, IQVIA, MSD, Roche; travel fees from AstraZeneca, Eisai, Servier; he is on the advisory board for AstraZeneca, Jazz, Eisai, Incyte, Roche. LR received consulting fees from AbbVie, AstraZeneca, Basilea, Bayer, BMS, Boehringer Ingelheim Eisai, Elevar Therapeutics, Exelixis, Genenta, Guerbet, Hengrui, Incyte, Ipsen, Jazz Pharmaceuticals, MSD, Nerviano Medical Sciences, Roche, Servier, Taiho Oncology, and Zymeworks; lecture fees from AstraZeneca,

Bayer, Biologix, BMS, Eisai, Guerbet, Incyte, Ipsen, Roche, and Servier; travel expenses from AstraZeneca and Servier; and research grants (to institution) from AbbVie, AstraZeneca, BeiGene, BMS Exelixis, Fibrogen, Incyte, Ipsen, Jazz Pharmaceuticals, MSD, Nerviano Medical Sciences, Roche, Servier, Taiho Oncology, TransThera, and Zymeworks. AC received consulting fees from Jazz Pharmaceuticals and AstraZeneca. TJK received consulting fees from Resolution Therapeutics, Clinnovate Health, HistoIndex, Fibrofind, Kynos Therapeutics, Perspectum, Concept Life Sciences, Servier Laboratories, Taiho Oncology, Roche, Jazz Pharmaceuticals; speaker fees from Servier Laboratories, Incyte Corporation, Jazz Pharmaceuticals, Astrazeneca, HistoIndex; he is on the advisory board for Servier Laboratories, Taiho Oncology, Roche and Jazz Pharmaceuticals, and he is a committee member of the Pathological Society of Great Britain and Ireland, Cholangiocarcinoma UK, UK Liver Pathology Group. CB received honoraria as speaker from AstraZeneca, Incyte, Servier, and honoraria as consultant from Incyte, Servier, Boehringer Ingelheim, AstraZeneca, Tahio, Jazz Pharmaceuticals, Molecular Partners, Delcath; received research funds from Avacta, Medannex, Servier, and her spouse is an employee of AstraZeneca. The other authors did not declare any competing interests.

Please refer to the accompanying ICMJE disclosure forms for further details.

Authors' contributions

Conceptualization: GT and CB; Formal analysis: GT and CB; Investigation: GT, JG, OK, HS, AA, SM, JM, HI, RM, SC, MH, AL, TJK, CB; Supervision: ACG, LR and CB; Resources: AC and CB; Visualization: GT and CB; Writing – original draft: GT, JG and CB; Writing – review and editing: all authors; Funding acquisition: CB.

Data availability

All data supporting this study are provided in full in the Results and in the Supplementary materials.

Acknowledgments

This publication is based upon work from the European Network for the Study of Cholangiocarcinoma (ENSCCA) and the COST-action Precision BTC Network (CA22125), supported by COST (European Cooperation in Science and Technology), www.COST.eu.

Supplementary data

Supplementary data to this article can be found online at https://doi.org/10.1016/j.jhepr.2026.101899.

References

Author names in bold designate shared co-first authorship.

[1] **Qurashi M**, **Vithayathil M**, Khan SA. Epidemiology of cholangiocarcinoma. Eur J Surg Oncol 2025;51(2):107064. https://doi.org/10.1016/j.ejso.2023.107064.
[2] Rimassa L, Khan S, Groot Koerkamp B, et al. Mapping the landscape of biliary tract cancer in Europe: challenges and controversies. Lancet Reg Health Eur 2025;50:101171. https://doi.org/10.1016/j.lanepe.2024.101171.
[3] Bridgewater J, Fletcher P, Palmer DH, et al. Long-term outcomes and exploratory analyses of the randomized phase III BILCAP study. J Clin Oncol 2022;40(18):2048–2057. https://doi.org/10.1200/JCO.21.02568.
[4] Primrose JN, Fox RP, Palmer DH, et al. Capecitabine compared with observation in resected biliary tract cancer (BILCAP): a randomised, controlled, multicentre, phase 3 study. Lancet Oncol 2019;20(5):663–673. https://doi.org/10.1016/S1470-2045(18)30915-X.
[5] Oh DY, He AR, Qin S, et al. Durvalumab plus chemotherapy in advanced biliary tract cancer: 3-year overall survival update from the phase III TOPAZ-1 study. J Hepatol 2025;83(5):1092–1101. https://doi.org/10.1016/j.jhep.2025.05.003.
[6] Oh DY, Ruth He A, Qin S, et al. Durvalumab plus gemcitabine and cisplatin in advanced biliary tract cancer. NEJM Evid 2022;1(8). https://doi.org/10.1056/EVIDoa2200015. EVIDoa2200015.
[7] Finn RS, Ueno M, Yoo C, et al. Three-year follow-up data from KEYNOTE-966: pembrolizumab (pembro) plus gemcitabine and cisplatin (gem/cis) compared with gem/cis alone for patients (pts) with advanced biliary tract cancer (BTC). J Clin Oncol 2024;42(16_suppl). https://doi.org/10.1200/JCO.2024.42.16_suppl.4093. 4093-4093.
[8] **Kelley RK**, **Ueno M**, Yoo C, et al. Pembrolizumab in combination with gemcitabine and cisplatin compared with gemcitabine and cisplatin alone for patients with advanced biliary tract cancer (KEYNOTE-966): a randomised, double-blind, placebo-controlled, phase 3 trial. Lancet 2023;401(10391):1853–1865. https://doi.org/10.1016/S0140-6736(23)00727-4.
[9] Tesini G, Ibrahim H, Rimassa L, et al. Evolving precision: updates in targeted therapy for cholangiocarcinoma. Hepatology 2025. https://doi.org/10.1097/HEP.0000000000001541.
[10] Vogel A, Bridgewater J, Edeline J, et al. Biliary tract cancer: ESMO Clinical Practice Guideline for diagnosis, treatment and follow-up. Ann Oncol 2023;34(2):127–140. https://doi.org/10.1016/j.annonc.2022.10.506.
[11] Vogel A, Ducreux M. clinicalguidelines@esmo.org EGCEa. ESMO Clinical Practice Guideline interim update on the management of biliary tract cancer. ESMO Open 2025;10(1):104003. https://doi.org/10.1016/j.esmoop.2024.104003.
[12] Malka D, Borbath I, Lopes A, et al. Molecular targeted maintenance therapy versus standard of care in advanced biliary cancer: an international, randomised, controlled, open-label, phase III umbrella trial (SAFIR-ABC10-Precision Medicine). ESMO Open 2025;10(5):104540. https://doi.org/10.1016/j.esmoop.2025.104540.
[13] Ikeda M, Kuang M, Oh DY, et al. 261TiP Randomized, open-label, multicenter, phase III study of trastuzumab deruxtecan (T-DXd) with rilvegostomig *vs.* standard of care (SOC) in first-line, human epidermal growth factor receptor 2 (HER2)-expressing, locally advanced or metastatic (LA/m) biliary tract cancer (BTC): DESTINY-BTC01. Ann Oncol 2024;35:S1501. https://doi.org/10.1016/j.annonc.2024.10.279.
[14] Harding JJ, El-Khoueiry A, Ueno M, et al. A phase 1b/2, safety lead-in and dose-expansion trial of ivosidenib plus durvalumab and gemcitabine/cisplatin as first-line therapy in patients with locally advanced, unresectable or metastatic cholangiocarcinoma with an IDH1 mutation. J Clin Oncol 2025;43(16_suppl). https://doi.org/10.1200/JCO.2025.43.16_suppl.TPS4219. TPS4219-TPS4219.
[15] Harding JJ, Macarulla T, Pant S, et al. HERIZON-BTC-302: a phase 3 study of zanidatamab with standard-of-care (SOC) therapy *vs.* SOC alone for first-line treatment of human epidermal growth factor receptor 2 (HER2)-positive advanced/metastatic biliary tract cancer (BTC). J Clin Oncol 2025;43(4_suppl). https://doi.org/10.1200/JCO.2025.43.4_suppl.TPS648. TPS648-TPS648.
[16] Bartley AN, Washington MK, Colasacco C, et al. HER2 testing and clinical decision making in gastroesophageal adenocarcinoma: guideline from the college of American pathologists, American society for clinical Pathology, and the American society of clinical Oncology. J Clin Oncol 2017;35(4):446–464. https://doi.org/10.1200/JCO.2016.69.4836.
[17] Harding JJ, Kim JW, Oh D-Y, et al. Concordance analysis between tumor tissue HER2 status by immunohistochemistry (IHC) and in situ hybridization (ISH) and a translational analysis of plasma ctDNA in patients (pts) with biliary tract cancer (BTC): an exploratory analysis from the phase 2 HERIZON-BTC-01 trial. J Clin Oncol 2025;43(16_suppl). https://doi.org/10.1200/JCO.2025.43.16_suppl.4102. 4102-4102.
[18] Silverman IM, Hollebecque A, Friboulet L, et al. Clinicogenomic analysis of FGFR2-rearranged cholangiocarcinoma identifies correlates of response and mechanisms of resistance to pemigatinib. Cancer Discov 2021;11(2):326–339. https://doi.org/10.1158/2159-8290.CD-20-0766.
[19] Hempel L, Lapa C, Dierks A, et al. A new promising oncogenic target (p.C382R) for treatment with pemigatinib in patients with cholangiocarcinoma. Ther Adv Med Oncol 2022;14:17588359221125096. https://doi.org/10.1177/17588359221125096.
[20] Vogel A, Sahai V, Hollebecque A, et al. An open-label study of pemigatinib in cholangiocarcinoma: final results from FIGHT-202. ESMO Open 2024;9(6):103488. https://doi.org/10.1016/j.esmoop.2024.103488.
[21] Lamarca A, Palmer DH, Wasan HS, et al. Second-line FOLFOX chemotherapy versus active symptom control for advanced biliary tract cancer (ABC-06): a phase 3, open-label, randomised, controlled trial. Lancet Oncol 2021;22(5):690–701. https://doi.org/10.1016/S1470-2045(21)00027-9.
[22] Abou-Alfa GK, Macarulla T, Javle MM, et al. Ivosidenib in IDH1-mutant, chemotherapy-refractory cholangiocarcinoma (ClarIDHy): a multicentre, randomised, double-blind, placebo-controlled, phase 3 study. Lancet Oncol 2020;21(6):796–807. https://doi.org/10.1016/S1470-2045(20)30157-1.
[23] Lee CK, Chon HJ, Cheon J, et al. Trastuzumab plus FOLFOX for HER2-positive biliary tract cancer refractory to gemcitabine and cisplatin: a multi-institutional phase 2 trial of the Korean Cancer Study Group (KCSG-HB19-14). Lancet Gastroenterol Hepatol 2023;8(1):56–65. https://doi.org/10.1016/S2468-1253(22)00335-1.
[24] Meric-Bernstam F, Makker V, Oaknin A, et al. Efficacy and safety of trastuzumab deruxtecan in patients with HER2-expressing solid tumors:

primary results from the DESTINY-PanTumor02 phase II trial. J Clin Oncol 2024;42(1):47–58. https://doi.org/10.1200/JCO.23.02005.
[25] Zhu AX, Macarulla T, Javle MM, et al. Final overall survival efficacy results of ivosidenib for patients with advanced cholangiocarcinoma with IDH1 mutation: the phase 3 randomized clinical ClarIDHy trial. JAMA Oncol 2021;7(11):1669–1677. https://doi.org/10.1001/jamaoncol.2021.3836.
[26] Kim Y, Jee S, Kim H, et al. EGFR, HER2, and MET gene amplification and protein expression profiles in biliary tract cancer and their prognostic significance. Oncologist 2024;29(8):e1051–e1060. https://doi.org/10.1093/oncolo/oyae076.
[27] **Xu H**, **Liang Y**, Tang W, Yang X, et al. Clinical significance of HER2 overexpression in biliary tract carcinoma –a meta analysis. Front Oncol 2025;15: 1534005. https://doi.org/10.3389/fonc.2025.1534005.
[28] **Kim H**, **Oum C**, Cho SI, et al. Artificial intelligence-powered human epidermal growth factor receptor 2 quantification and clinical outcomes in human epidermal growth factor receptor 2-positive biliary tract cancer treated with trastuzumab plus folinic Acid, fluorouracil, and oxaliplatin. JCO Precis Oncol 2025;9:e2500510. https://doi.org/10.1200/PO-25-00510.
[29] Bouattour M, Valle JW, Vogel A, et al. Characterization of long-term survivors in the TOPAZ-1 study of durvalumab or placebo plus gemcitabine and cisplatin in advanced biliary tract cancer. J Clin Oncol 2023;41(4_suppl). https://doi.org/10.1200/JCO.2023.41.4_suppl.531. 531-531.
[30] Oh D-Y, Qin S, Antonuzzo L, et al. Analysis of clinically actionable alterations in baseline tumor versus plasma samples in participants of the TOPAZ-1 study of durvalumab plus gemcitabine and cisplatin in advanced biliary tract cancer. J Clin Oncol 2025;43(4_suppl). https://doi.org/10.1200/JCO.2025.43.4_suppl.625. 625-625.
[31] Sulkowski PL, Oeck S, Dow J, et al. Oncometabolites suppress DNA repair by disrupting local chromatin signalling. Nature 2020;582(7813):586–591. https://doi.org/10.1038/s41586-020-2363-0.
[32] Molenaar RJ, Radivoyevitch T, Nagata Y, et al. IDH1/2 mutations sensitize acute myeloid leukemia to PARP inhibition and this is reversed by IDH1/2-mutant inhibitors. Clin Cancer Res 2018;24(7):1705–1715. https://doi.org/10.1158/1078-0432.CCR-17-2796.
[33] **Sulkowski PL**, **Corso CD**, Robinson ND, et al. 2-Hydroxyglutarate produced by neomorphic IDH mutations suppresses homologous recombination and induces PARP inhibitor sensitivity. Sci Transl Med 2017;9(375). https://doi.org/10.1126/scitranslmed.aal2463.
[34] Doroshow DB, Wei W, Mehrotra M, et al. Platinum sensitivity in IDH1/2 mutated intrahepatic cholangiocarcinoma: not all "BRCAness" is created equal. Cancer Invest 2023;41(7):646–655. https://doi.org/10.1080/07357907.2023.2242957.
[35] Niger M, Nichetti F, Casadei-Gardini A, et al. Platinum sensitivity in patients with IDH1/2 mutated *vs.* wild-type intrahepatic cholangiocarcinoma: a propensity score-based study. Int J Cancer 2022;151(8):1310–1320. https://doi.org/10.1002/ijc.34182.
[36] Liao P, Cao L, Chen H, et al. Analysis of metastasis and survival between extrahepatic and intrahepatic cholangiocarcinoma: a large population-based study. Medicine (Baltimore) 2021;100(16):e25635. https://doi.org/10.1097/MD.0000000000025635.
[37] Nimish T, Gonzalez T, Nano O, et al. Extrahepatic cholangiocarcinoma versus intrahepatic cholangiocarcinoma: a comparison of survival outcomes and use of palliative care in diagnosed patients. J Clin Oncol 2022;40(16_suppl). e16192-e16192.

Keywords: biliary tract cancer; targeted therapies; *IDH1* R132 mutations; HER2 overexpression.
Received 13 December 2025; received in revised form 20 April 2026; accepted 10 May 2026; Available online 20 May 2026

Journal of Hepatology, Volume *8*

Supplemental information

IDH1 R132 mutations or HER2-positivity and benefit from platinum-based therapy for biliary tract cancers

Giulia Tesini, Jack Greaves, Olivia Knight, Holly Shand, Angela Ammirabile, Sophie McHaffie, Jen Milne, Halima Ibrahim, Rosemary Meenan, Sophia Campbell, Margaret Henderson, Andrea Lampis, Louis Chesler, Andrea Casadei-Gardini, Lorenza Rimassa, Alan Christie, Timothy J. Kendall, and Chiara Braconi

IDH1 R132 mutations or HER2-positivity and benefit from platinum-based therapy for biliary tract cancers

Giulia Tesini, Jack Greaves, Olivia Knight, Holly Shand, Angela Ammirabile, Sophie McHaffie, Jen Milne, Halima Ibrahim, Rosemary Meenan, Sophia Campbell, Margaret Henderson, Andrea Lampis, Louis Chesler, Andrea Casadei-Gardini, Lorenza Rimassa, Alan Christie, Timothy J Kendall, Chiara Braconi

Table of contents

Fig. S1……………………………………………………………………………………….2

Fig. S2……………………………………………………………………………………….3

Fig. S3……………………………………………………………………………………….4

Table S1……………………………………………………………….……..separate excel file

Table S2…………………………………………………………………...separate excel file

Table S3……………………………………………………………….……..separate excel file

Table S4…………………………………………………………………………………….5

Supplementary figures

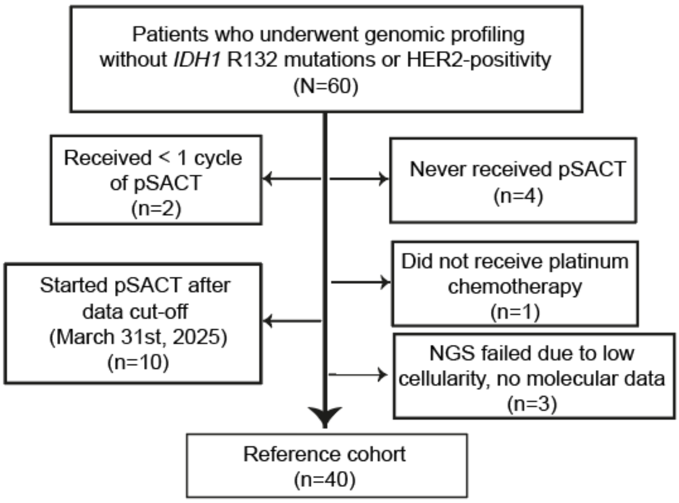

Fig. S1. CONSORT diagram of patients included in the reference cohort.

Abbreviations: NGS, Next-Generation Sequencing; pSACT, palliative systemic anti-cancer treatment.

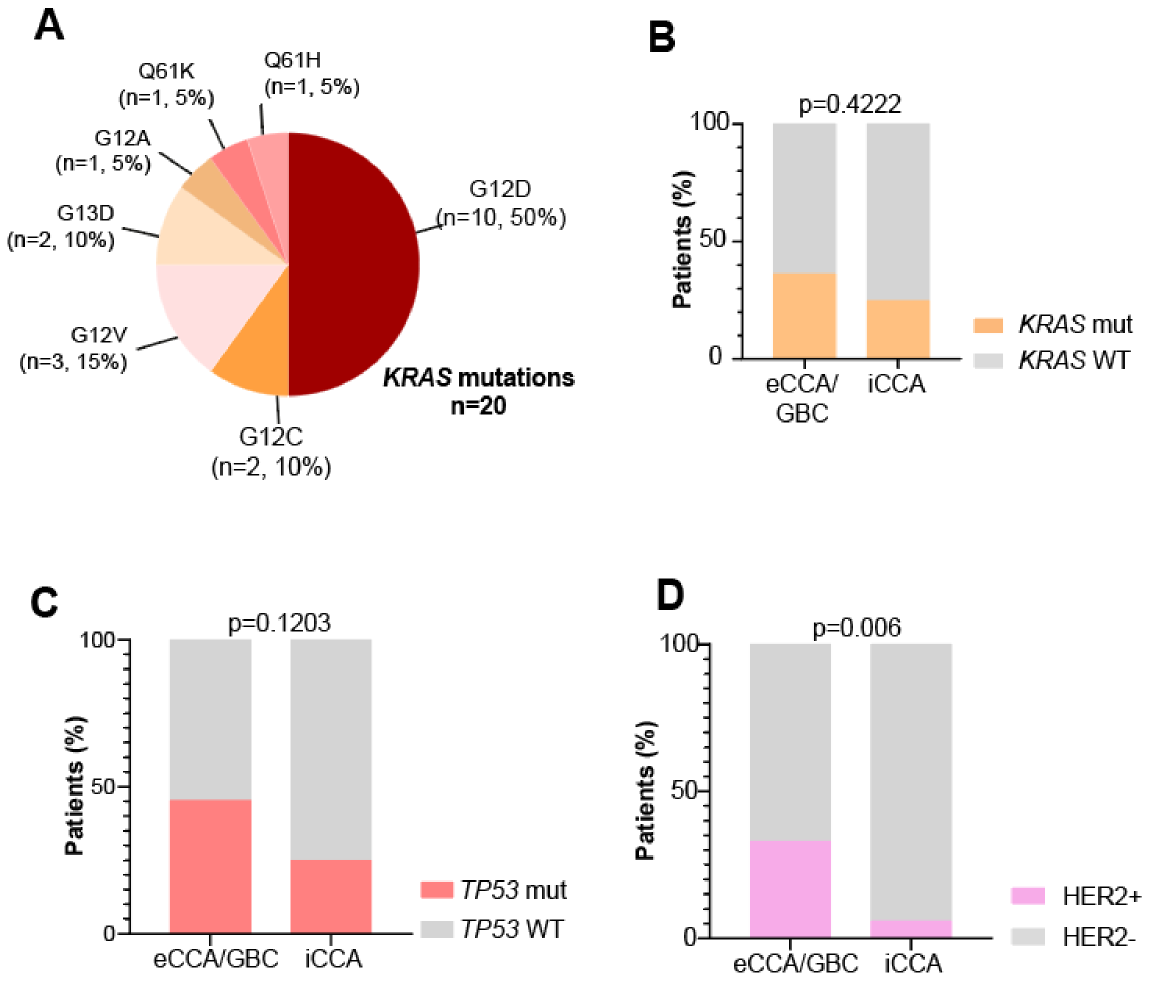

Fig. S2. Frequency of *KRAS* mutations (A) and distribution of the most common molecular alterations in our cohort across BTC subtypes (*KRAS* mutations – B; *TP53* alterations – C; HER2 overexpression (IHC 3+) or amplification (FISH-positive) – D). Variables have been compared using Fisher's exact test.

Abbreviations: eCCA, extrahepatic cholangiocarcinoma; GBC, gallbladder cancer; iCCA, intrahepatic cholangiocarcinoma; mut, mutated; WT, wild-type.

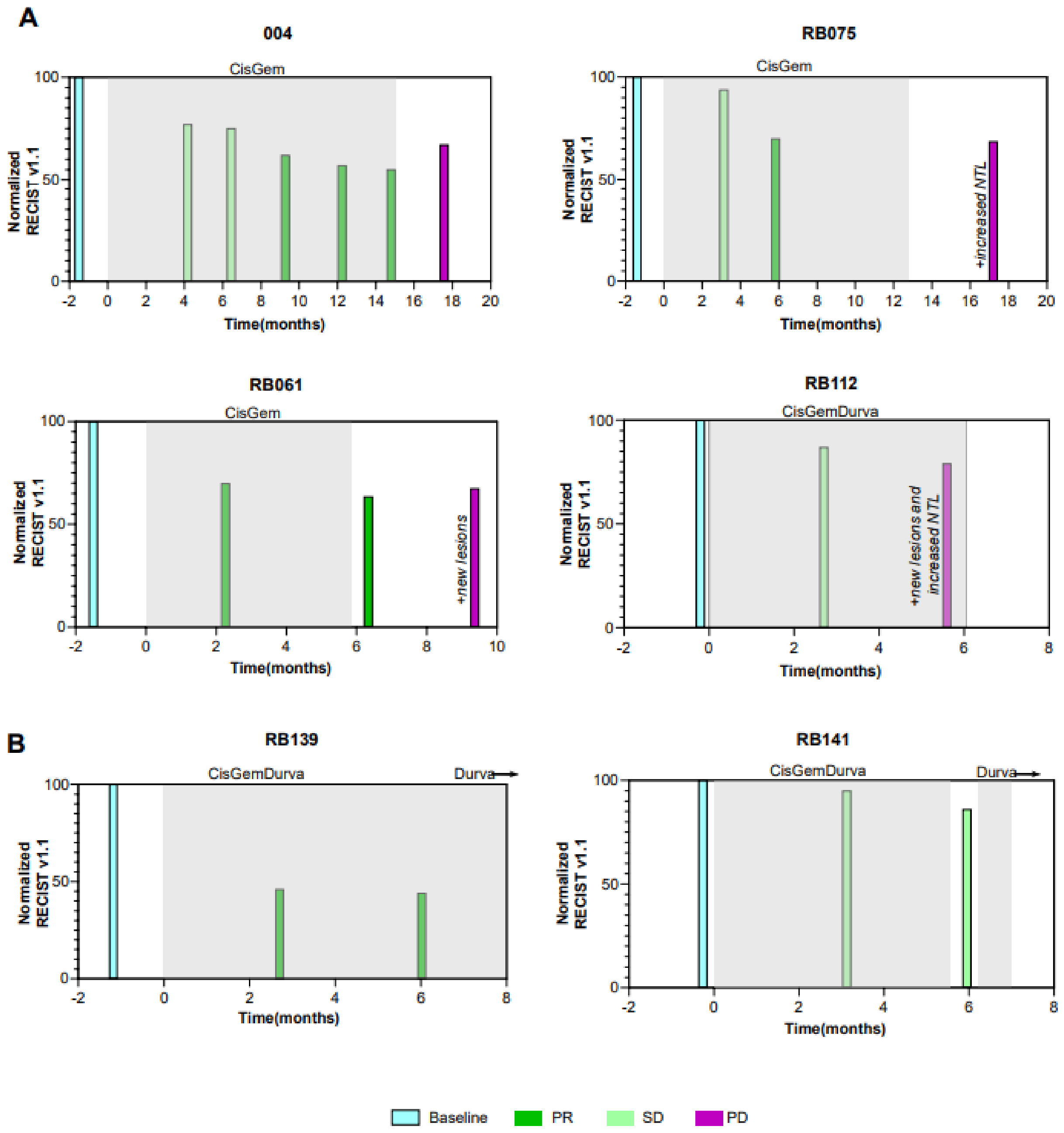

Fig. S3. Radiological response to treatment according to RECIST v1.1 criteria in patients with IDH1 R132 mutations. (A) Patients who have already progressed to first-line pSACT. (B) Patients who are still on treatment as of September 30th, 2025 (data cut-off). Radiological response improves over time and maximum radiological response is achieved > 5 months from treatment start in 5 cases. Abbreviations: CisGemDurva, cisplatin-gemcitabine-durvalumab; NTL, non target lesion; PD, progressive disease; PR, partial response; pSACT, palliative systemic anti-cancer treatment; SD, stable disease.

Supplementary tables

Table S1, S2 and S3 are provided in a separate Excel file.

Table S4. Baseline characteristics of the reference cohort.

	**N=40**
**Female, n(%)**	16 (40)
**Median age at diagnosis, years (IQR)**	62 (57-72)
**BTC subtype, n(%)**	
iCCA	19 (47.5)
pCCA	8 (20)
dCCA	6 (15)
GBC	7 (17.5)
**Resectable disease at diagnosis, n(%)**	12 (30)
**First-line pSACT, n(%)**	
Platinum/gemcitabine	14 (35)
Platinum/gemcitabine/durvalumab	26 (65)
**Median CA19-9 at start of 1L pSACT, kU/L (IQR)**	280 (33.5-3665)
**Median CEA at start of 1L pSACT, ug/L (IQR)***	3.5 (2.05-11.5)

*CEA at baseline not dosed for 3 patients

Abbreviations: 1L, first line; BTC, biliary tract cancer; CA19-9, carbohydrate antigen 19-9; CEA, carcinoembryonic antigen; dCCA, distal cholangiocarcinoma; GBC, gallbladder cancer; iCCA, intrahepatic cholangiocarcinoma; IQR, interquartile range; pCCA, perihilar cholangiocarcinoma; pSACT, palliative systemic anti-cancer treatment.
